# Supplementary material for: Healthcare systems barriers and strategies for pre-exposure prophylaxis utilization amongst young females in Gauteng province: Registered nurse’s perspectives
Source: PLoS One. 2025 Jun 12;20(6):e0294182. doi: 10.1371/journal.pone.0294182 (PMC12161546; doi:10.1371/journal.pone.0294182)
Supplement: S1 File — This is SI Fig transcribed and analysed focus group discussion data for registered nurses. (PDF) [file pone.0294182.s001.pdf]

Project: Doreen Onkarabile Mudau

Focus Groups Discussion

Report created by Prof. Anna van der Wath on 2022/04/21

Quotation Report – Grouped by: Codes

All (365) quotations

○ Perceived barriers to PrEP uptake and retention among females: 2.1 Health care system barriers. Barriers to access PrEP

7 Quotations:

3:31 ¶ 287 – 289 in Clinic B - FGD

PARTICIPANT 38 What I've noticed ... Others they don't want to do it. Even if they've got everything, they'll just say there's no sister doing it. Like, should we mention the names of the clinic.

INTERVIEWER: Ja, don't mention clinics.

PARTICIPANT 37 Ja, like clinic A they'll just say the sister is no here.

3:32 ¶ 291 in Clinic B - FGD

PARTICIPANT 37 And tried to send one sister there to be there on days. If the sister is not there on that day all the clients will be turned back. Or sent to otherBecause the sister is not there.

3:33 ¶ 296 in Clinic B - FGD

PARTICIPANT 36 The list that was designated that all the clinics are offering so wena I was surprised to say why are they still referring to us if those clinics are still ...

4:39 ¶ 90 in Clinic C - FGD

PARTICIPANT 43 …forgetting that there is PREP. Until a person necessarily comes in. Like now, we have got a challenge, the PREP room is closed. Like she said, the Hanover sister is gone. We see all those people that were coming in, who were taking PREP, now they are being decentralised everywhere. And then you come, she will come in, and then we have got something that is called the catchment area. Like you said, it was offered in five facilities, alright. Okay, the person is staying wherever and that is one of the reasons

that makes it even more difficult to continue with care, when it comes to PREP, because of maybe monetary problems, or whatever, or that catchment thing, that has happened. She will come to Lilian Ngoyi where she has been taking PREP from B1 for five years, or whatever. Now she comes to you, as a person that because you have not been exposed to such for a long time, when the person comes, he comes, for argument's sake. The first thing that you see is the address, you don't look at the youth, you say, the person must go to Pimville. And maybe the Pimville has got that stigma that they are talking, that maybe many of my friends are going there and I do not want to go there, I want to go to Clinic A for personal reasons. That person will be lost to the system, because at the end of the day, she will not make that place that she needs to go to. But because of, I think maybe the main problem is shortage of staff, that most of, like now so many people have gone, so many departments have been closed because of shortage of people who are rendering the services. And that is the most challenging issue that we face.

4:71 ¶ 214 in Clinic C - FGD

PARTICIPANT 39I am not sure now, right now, but I think all the clinics are offering now. The challenge now is if a majority of people, let us say people who are staying in Orlando, where initially we started PREP here, in this clinic. Now, Pimville has PREP and Orlando has PREP and so on. And then you get somebody who will say, no, you stay in Pimville sisi, you have to go back to your clinic. And the client will say, no, but sister, I started here, I cannot go back, like participant six was saying. They get used to you, but I cannot give it, she is not there, I am new and then you have to go back, your clinic offers the same service. And that young person will say, alright fine, let me go home.

4:74 ¶ 222 – 223 in Clinic C - FGD

PARTICIPANT 45 Participant zero. So, look guys , I am still new in the Department, yes, it is my first year being a PEC. So, what I have noticed with the patient with HIV, especially, some of them, they do not have the right addresses, even if you ask them, those addresses on the outside of their file, they would say they do not even know their numbers of their addresses. Because they are afraid to go to the nearest place, because they are scared of those people who live around them, to be seen or all those things. And even

with PREP also, I think because we were talking about the stigma of HIV and PREP, they will be given the same problem with the HIV. People will want you to go far away for their PREP. So I am not sure how can we address those things of catchment areas and stuff like that.

5:27 ¶ 99 in Clinic D - FGD

PARTICIPANT 46: You know the challenges that I got from them it is the baseline blood, it is that? immediately if you know that you are not sick, it is up to you, it is your decision to do whatever. That is why I have seen from them to say okay fine Sister I will start PrEP today. Okay and then you explain that I am going to send you to our blood room and do bloods and then they will tell you no, no I cannot. So we are going back to that issue of saying that they are scared of needles. So that is the other challenge that we get from them. So they refuse it. Immediately when you tell them about you are going to take baseline

blood, they do not, they refuse. Even if you try to explain they will say, no I, let me go and think about it and I will come back.

○ Perceived barriers to PrEP uptake and retention among females: 2.1 Health care system barriers. Contra-indications for PrEP initiation

7 Quotations:

1:43 ¶ 176 – 182 in Clinic A - FGD

PARTICIPANT 287: But we find that when they come into the room especially to find information, it is totally different information, they do not give them the correct information because they are pushing stats, statistics for the NGOs.

INTERVIEWER: So what makes it different? So what difference have you picked up?

PARTICIPANT 287: Monday I had about 5 school kids who were brought by a counsellor to say I must initiate this PrEP. Only one of them was eligible because they all state we wanted to test for HIV, why do you want to test for HIV, they said I must test for HIV so I can be on PrEP so I can prevent HIV. Okay and I left them to be alone, when I asked are you sexually active because one of the criteria is you must be sexually active, no I am still a virgin but I want PrEP.

INTERVIEWER: Why do you want PrEP?

PARTICIPANT 287: Ja why do you want PrEP?

PARTICIPANT 28: In case.

PARTICIPANT 287: In case, it is just in case [and when are you going to start because it is just in case, I do not know Sister, but just in case.

1:44 ¶ 184 – 186 in Clinic A - FGD

PARTICIPANT 28: But one of the criteria is that you must be sexually active. So you can search with your eyes and they are searching with their …

INTERVIEWER: With what, searching with what?

PARTICIPANT 28: With the thing. There is difference when you are having sex, contact like that and if you are still a virgin you are not being touched-

1:45 ¶ 190 in Clinic A - FGD

PARTICIPANT 287: I am going to give you, there is that part that says, are you injecting drugs, if it is yes, then I would give you, but if it is all no, then why should I give you.

1:46 ¶ 191 in Clinic A - FGD

PARTICIPANT 29: But when it comes to an accident that is just something that, it is there at back. We cannot give you PrEP because you are thinking you might be intimate next time at some stage of your life. So if you are not sexually active and you are not using drugs, do we really need to give you PrEP?

5:39 ¶ 160 in Clinic D - FGD

PARTICIPANT 47: I am going to add that children under 15 they cannot take TDF, so for them they have to wait until they are 15 and above, because I am not sure if it is the latest. I was trained on the first one, but that first one was 40 kg and 15.

6:42 ¶ 121 in Clinic E - FGD

PARTICIPANT 53: You know for that I am confused, because they will tell you that okay, when we went for training they said it is only for youth, from 15 years to 24 years and then after they said okay if you come across that the partner is positive, you are negative, so you can remain negative, you must take PrEP, in that case I am not sure if there is guidelines or what for older people.

6:43 ¶ 125 in Clinic E - FGD

PARTICIPANT 54: So yes, with regards with eligibility like I said before, it is for everyone that is HIV negative at this point, unlike before when it used to be just for youth, just for a person who is HIV positive, just for men who sleep with men or prostitutes, now for everyone that is HIV negative, they can get it.

○ Perceived barriers to PrEP uptake and retention among females: 2.1 Health care system barriers. Ineffective workflow and staff shortages

16 Quotations:

1:51 ¶ 222 in Clinic A - FGD

PARTICIPANT 287: Okay, and even if you can try to cramp it down knowing [that I am going to have these 5 or 10 people who may like to take PrEP at this moment for you as a health professional it is also exhausting to be saying the same thing to this one and that one, it is the same, word by word to that one so when they leave they are leaving with the very same information.

1:53 ¶ 232 in Clinic A - FGD

PARTICIPANT 287: And yet at the same time you as a health professional you end up being drained talking of the same thing, same.

1:97 ¶ 372 in Clinic A - FGD

PARTICIPANT 287: So you can see a client after 2 then you cannot initiate. You cannot initiate because you tell that patient they will be like, no Sister, tomorrow I have got exams.

1:133 ¶ 366 in Clinic A - FGD

PARTICIPANT 28: You have got a patient, got all the tubes and everything, then they say no we cannot take blood now, the guy who fetches the blood is not coming today. That means the patient will have to come back tomorrow, then they will not come back.

3:29 ¶ 245 in Clinic B - FGD

PARTICIPANT 38 these kids they're always in a hurry. And it's too much work, like when, you need to give into that you have the medication, you need to take history, you need to do bloods, others they also do family planning and they are sick at the same time.

3:30 ¶ 251 in Clinic B - FGD

PARTICIPANT 33 It makes them not to take PrEP. They will just go out and if they come for family planning, because we recruit from the family planning ones, so when you're still busy with the one whose interested they will move out and go to the family planning side and go.

4:45 ¶ 154 in Clinic C - FGD

PARTICIPANT 44 Yes, it affects everything so much, because in the morning if you are taken from point A to point Z, from point Z to point Q to point M, you do not know where you are, you do not even know where you belong. You even console yourself by telling yourself, alright…

4:53 ¶ 388 in Clinic C - FGD

PARTICIPANT 43 I fully agree. We need to be empowered. It is like they expect too much from us, to a point that we are even having burnout from the programmes that are . Like when we started, I said, we even forget to offer PREP sometimes, because of the things that we are expected to do. So, l fully agree with her.

4:62 ¶ 244 in Clinic C - FGD

PARTICIPANT 40: Not that we do not want to, but we do not have time, due to the large numbers of patients that we see. You do not have time to sit with one patient for more than ten minutes, looking at the queue outside. Yes, and then, somewhere, somehow, we are depriving our patients, but …

4:83 ¶ 258 in Clinic C - FGD

PARTICIPANT 42 I agree with them, even the that are supposed to be helping, they are allocated outside. They screen for TB outside the clinic. They are doing COVID tests outside the clinic, so they cannot assist us inside. So, there is just nobody to give health education.

4:84 ¶ 264 in Clinic C - FGD

PARTICIPANT 42 Hence, they end up with that little information, not enough. Because I think this person needs a lot of time to reinforce some of the things. But that time is not there.

5:29 ¶ 105 in Clinic D - FGD

You know the other thing that they complain about; [it is that days are not the? same at the clinic. They will come and then maybe when they come that day it is a hectic day, it is not like you are only going to focus on her or him. They are lazy of queuing; some, is it not that sometimes you go to the community and we initiate them in the community and then you expect them their next visit to come to the nearest clinic and then they do not even want to come to the clinic. And then some they will say, no if I go to the clinic, my the community will say why did you go to the clinic and then now it becomes a stigma he or she attends the clinic you see. So those are their….

6:21 ¶ 62 in Clinic E - FGD

PARTICIPANT 53: that case, [when it comes to shortage of staff because sometimes you will see Sister she is alone working and then she is doing the pregnant women and all the things, so she had to send the patient to me, to Connie or to Sister it is where the patient is going to default and then she will leave the file and go and nothing we can do because we are having the shortage and then we are trying for them not to wait for a long time, but if you send her the patient, they end up leaving.

6:26 ¶ 78 in Clinic E - FGD

PARTICIPANT 54: Okay, so because where we are, there is not only, so I feel like every person should be actually going to where their address, that way if you are having a patient going to their address is here and they are going to the clinic nearest to them, it will be better, because then they will not have one being congested with all the patients all over or around the area coming to one clinic, you understand, so at the end of the day, even if we do have staff, if we are already having all the patients coming to one place, then we are going to also have a problem, because the queue, that will mean it will have a lot of people there, but we can always have more patients than the staff that is hired, so we really also need to look at the location of the patient.

6:27 ¶ 84 in Clinic E - FGD

PARTICIPANT 53: Yes and she is telling the point, it is true, they need to go their respective place, like demarcation, if we can work for demarcation because we have more than four clinics here around this clinic, but everyone will want to come to this Clinic. End of the day we are not affording to do what we are supposed to do because of large numbers that we are supposed to see to.

6:47 ¶ 138 in Clinic E - FGD

PARTICIPANT 53: I agree with them, yes, shortage of staff, it is a problem, all departments, we cannot be effective while we are having the challenge of shortage, yes, because we are pushing the line.

○ Perceived barriers to PrEP uptake and retention among females: 2.1 Healthcare system barriers. Unavailability of nurses dedicated to provide PrEP services to youth

14 Quotations:

1:79 ¶ 308 in Clinic A - FGD

PARTICIPANT 28: Especially if they want a specific person, I am working with PARTICIPANT 287, you know what they say? I want the Sister who was next door, they want that Sister and they work for you.

1:80 ¶ 312 – 314 in Clinic A - FGD

PARTICIPANT 28: With one healthcare professional. They will say no, she is not here, okay, bye, I will come back .

PARTICIPANT 287: I will come back when she comes back.

PARTICIPANT 28: And they leave.

1:122 ¶ 318 in Clinic A - FGD

PARTICIPANT 30: and also I guess once you are attached or assimilated to one professional because this person understands me or we have built a rapport with the professional they sometimes want to see the same person. So that is what discourages them from coming back.

3:13 ¶ 105 in Clinic B - FGD

I think it would be negatively affected the programme because it means the person for one reason or the other is not there then the problem is holding. Is not continuing. And then also to decline for coming, remember they are attending school or whatever. For the very

appointment there that they take some time it’s a set back to them because on the other side you are sick, you are going every now and then to the clinic. It can come as a setback. She made the time to come, the next thing the nurse is not there so it’s really negatively affecting the programme.

3:54 ¶ 498 – 500 in Clinic B - FGD

PARTICIPANT 37 And another thing when they came, if the door is locked the go away, even if they have got them somewhere but if they find the door locked they went back and don’t take PrEP.

INTERVIEWER: It explains the whole thing.

PARTICIPANT 37 Even for like the follow ups, I think they will know this room is doing PrEP. When they come straight from the gate they know where they are going. So if there is no one there, they don’t even ask, they just turn back and they will come back. Because I came and there was no one, and then they went back.

3:67 ¶ 496 in Clinic B - FGD

PARTICIPANT 37 mentioned earlier on that a few people were chained on it, so honestly if I'm not send the person or the person lands up giving, I'm not going to assist. So maybe like also to add time as she said she was on maternity leave, she was the one running the program. So automatically PrEP so you better come back the other day. That could impact also why they were

4:18 ¶ 33 in Clinic C - FGD

PARICIPANT 44: ...another thing, the challenges which we have, these shortages really have a negative impact on this work, because they come and you are not there and they see that you are a sister and they want you. They call us by names, the and they say, you know works here, where is she? They turn up. They go back, you tell them to go into that room, there is somebody there. So, that affects them a lot of these changing of staff, for example, the sister was doing PREP, they will ask the sister, who I worked with, the contract is terminated from Hanover. These kids are so frustrated, because they were attached. So, it affects them a lot, really. It makes people not want to comply

4:21 ¶ 45 in Clinic C - FGD

PARTICIPANT 43 Yes, like she said, like changing healthcare professionals also, it will have a negative impact on them because now they develop a relationship with you.

4:28 ¶ 118 in Clinic C - FGD

So, I think utilisation of human resources is important there. When we train in a department, like youth, it needs certain, like maybe you train three people. These ones are going to do this in this department. Like we said, she worked there, participant six worked there. She understands the challenges of this youth. This youth wants somebody that they

are used to. So, what is the importance of training all of us? Today this youth comes, she finds me, tomorrow she will find this one. They are not comfortable, they are too sensitive. They want somebody that they can relate to, at least maybe two or three that they are used to. I think that would encourage compliance, but if we are going to change, today this one, tomorrow another, they will not come.

4:30 ¶ 47 in Clinic C - FGD

PARTICIPANT 43 They want a focal person. And that is the problem, again, I think in our clinics because we cannot be working in the same spot forever, one. Number two, again because of the companies that come in and out, they make a relationship with particular people, and then when you come in, you are a stranger.

4:54 ¶ 386 in Clinic C - FGD

PARTICIPANT 39Enough staff. The Department will introduce new programmes, but they will never hire. We have to stretch ourselves, the people who are in the system, we stretch ourselves to cater. Like they said, it is a programme that needs consistency. But if there is no, there is not that person who is, there is shortage. So, the patient will end up coming here, others are brought here, others wherever, whatever, whoever is helping the counsellors. So, when new programmes are introduced, can the Department hire new people?

4:64 ¶ 88 in Clinic C - FGD

PARTICIPANT 43 As much as we would like to help introduce PREP, I do not think that many young people are exposed to us, if I may say. What I am saying is that even if we did integration of services, because if it was done … we are urging everybody, when a patient is coming in and she is offered PREP, it would be easy. But unfortunately, it is not done like that. Like they used to go to a certain place where they are seen by a particular person, like she was saying, she was working in a particular room, and then she was the one who was doing it. And then most of us, we do forget that we need to offer. Like you would see this young person, but because you are not-

4:72 ¶ 218 in Clinic C - FGD

PARTICIPANT 39Especially if you have to transfer those who came initially. You have to transfer them back to their clinics, facilities. They feel no, they cannot, they love their new sister, they enjoy being here, at that particular, but again, because of their addresses, they have to go back. So, some of them will just leave the programme.

4:73 ¶ 220 in Clinic C - FGD

PARTICIPANT 39I would not use force, as such, it is the police that says stay in a particular area and there are services that are offered at your nearest clinic. The facility is allowed to give you a transfer to that, but because there are attachments, you know, emotional and all that.

○ Perceived barriers to PrEP uptake and retention among females: 2.2 Health care provider barriers. Age, gender and competency barriers

23 Quotations:

3:34 ¶ 319 in Clinic B - FGD

PARTICIPANT 36 To see Boetie for me to be confident to issue something I need to be confident to say okay I've got information I know what to do and I'm confident ... That I'm not making a mistake. So now you just pilot, you're just distributing and expecting me to do it, no I'm not going to do it, send them to Zola because they've received ...Some information.

3:40 ¶ 357 in Clinic B - FGD

PARTICIPANT 34 No. I have seen the was given by one of the sisters in our one department in the clinic and then I just read it this combination of 1, 2 and then I got that little information that you will take it to prevent yourself from but all this I don't have the details.

3:43 ¶ 371 in Clinic B - FGD

PARTICIPANT 38 Not trained, what I know is that it prevents HIV, that's all I know.

3:44 ¶ 367 in Clinic B - FGD

PARTICIPANT 32: Okay, I have an idea what PrEP is, but I wasn't trained so …

3:60 ¶ 434 in Clinic B - FGD

PARTICIPANT 34 No, I said what she is saying because the way she is asking me a patient and I don't have information and that's the good side. Right I give information here and then they are read after but I have given them personal information everything, they come with information. She is asking the questions I can't answer it. Then now I want you to just.

3:61 ¶ 436 in Clinic B - FGD

PARTICIPANT 32: If I can't answer some of the questions, then I say just go that side.

4:13 ¶ 92 in Clinic C - FGD

PARTICIPANT 39You know what? I am going to say this again and again. These people are being, new programmes are introduced, a small group of people are in service, they are

taught and that is it. People go on night duty, they go on leave, they come back and nobody cares. It is like participant six is saying, she was not trained, she must just go to the youth clinic. Nothing, no in-service, nothing. You learn on the way.

4:26 ¶ 108 in Clinic C - FGD

PARTICIPANT 43 We asked, actually, it was the beginning of COVID, last year, and then they told us that doctor whoever is not available to teach, because he cannot be meeting with a number of people. So, they could do virtual training. Unfortunately, we do not have such a facility, unless we are all connecting, we are on our phone. So, it was never done.

4:27 ¶ 112 in Clinic C - FGD

PARTICIPANT 42 Yes, I was trained. I did attend training for two days, but it was a very long time ago and after that, I did not work with those children. So, I did not even know if I had to go there, what to do.

4:29 ¶ 47 in Clinic C - FGD

PARTCIPANT 43: Then again, the age gap again is a problem. I think if it could be run by somebody young, who is almost their age. I mean, it is difficult for a sixty-year-old to talk sexual intercourse with a fifteen-year-old.

4:31 ¶ 49 in Clinic C - FGD

PARTICIPANT 43 Yes, I mean culturally it is not acceptable. Within your house you cannot be talking sex with a young person. That is how it is supposed to be. But we need to focus again on that.

4:41 ¶ 126 in Clinic C - FGD

PARTICIPANT 39Because now we work on Saturdays and Sundays and night duty. You can get that young person, at night, coming in with a burst condom, her focus is PEP. You have the knowledge, you sit down, you know there is this tablet, it works like this, blah-blah-blah. After this, you take … you know, even on Saturdays, Sundays, they come for whatever they are coming for. They stay in different areas. You give her education, based on the knowledge that you have. Even when they come up with questions, because they Google and know more than you. So, if you do not know your story, you are not-

4:66 ¶ 178 in Clinic C - FGD

PARTICIPANT 43 I do not know anything about that.

4:67 ¶ 182 in Clinic C - FGD

PARTICIPANT 42 I remember when I went for training, they were saying fourteen days.

4:68 ¶ 194 in Clinic C - FGD

PARTICIPANT 42 I will just take a guess and say twenty-eight days.

4:69 ¶ 196 in Clinic C - FGD

PARTICIPANT 43 I do not know.

5:32 ¶ 131 in Clinic D - FGD

PARTICIPANT 51: I understand. See those that are trained, we know very well we cannot, there are certain, there is a certain way that we need to deal with these young ones, so I think their attitude comes from that side. Obviously those who are not trained.

5:35 ¶ 243 in Clinic D - FGD

PARTICIPANT 46: I am also echoing on the training of all the medical staff. When I say medical staff I am not only saying the professional nurses and the clinicians. I am also saying the doctors as well they should also be trained on this and then also supporting the accessibility because I think some of the clinics there, it is not accessible because now, most of the medical, in that clinic the medical staff is not trained. That is where it is not accessible.

6:12 ¶ 23 in Clinic E - FGD

PARTICIPANT 54: because now there was this one patient coming in, talking about how they were with a nurse and the nurse was like, she was asking the nurse about PrEP because she had PrEP, so she was asking the nurse and then the nurse was like huh, this is ARV’s so why are you taking ARV’s, so the patient was like I just got scared and I just threw them away. You understand, she said she just threw them why are they giving me ARV’s whereas I am HIV negative, so we also not only the mothers or the child that actually need teaching or education or knowledge, but we also as clinicians we also have to have this information.

6:13 ¶ 25 in Clinic E - FGD

PARTICIPANT 54: And it also depends on whoever is giving the education when it comes to PrEP, because our people will also tell the patients that, but then again why use PrEP because it is making you, how can I say, to actually getting HIV, so by the time you actually get HIV, what if the pill, ARV’s, the actual TLB’s will not work because of you have already told your immune system that you have taken this tablets.

6:15 ¶ 33 in Clinic E - FGD

PARTICIPANT 56: No, I understand here in our facility, we are having a challenge especially with the clinicians, especially with the doctors, sometimes they do not even check the result, if the patient is requesting, [they hear about like they just initiate without testing. So as we are clinicians here, if we can stress more about the training, even the staff, yes, it will help.

6:16 ¶ 37 in Clinic E - FGD

PARTICIPANT 53: We will only have the problem like, we will only had the problem from the pharmacy, the patient came without testing, without creating a result or anything, they just prescribed one day for PrEP. Yes.

6:44 ¶ 129 in Clinic E - FGD

PARTICIPANT 54: to be initiated, also sometimes I would, like they said we do need updating, because one minute I am hearing we can initiate pregnant women, the next day it is like no, they are not eligible to actually get it, so we have to stop, so if you have started a patient on PrEP because they were HIV negative and all of that, now, pregnancy tests come positive, then you must stop it. So now I am also confused with that, so knowledge, updating will also help.

○ Perceived barriers to PrEP uptake and retention among females: 2.2 Health care provider barriers. Ethical dilemmas

19 Quotations:

1:52 ¶ 222 – 224 in Clinic A - FGD

PARTCIPANT 28: Whereby also let us say now you can do a group health talk, what happens to where now PARTICIPANT 30 is having a private kind of a question that does not need others to hear, even though it may be of good use for the rest to hear. She will also be looking at herself and saying that if I can be asking that question of which is personal to me, but yet at the same time that means I can end up being judged on what I have voiced out. You must also

INTERVIEWER: Okay so you are saying …

PARTICIPANT 287: We can do a group discussion pertaining to that, but if there is something that I would like to ask as a client and maybe it can benefit others, at that point you can be looking that this one will end up judging me with what I have just asked.

1:56 ¶ 245 in Clinic A - FGD

PARTICIPANT 30: I am just looking at their maturity level. This is someone who still has a guardian. Yes we know that they are already sexually active and unfortunately for us to turn someone away is not right, because they are already sexually active. It discourages me but it does not mean that I would not give them.

1:57 ¶ 247 in Clinic A - FGD

PARTICIPANT 30: Yes because I know that there is accountability there. There is someone who is older who can still support and remind this person, who at some point a 15-year-old is still reminded 'did you do your homework', you still need to go 'are you studying for exams' so then what are we saying about the level of maturity for someone to be accountable to take a pill each and every day.

1:61 ¶ 259 in Clinic A - FGD

PARTICPANT 29: What is discouraging it now goes into the values, morals and social beliefs as human beings generally that a 15-year-old is a child, when you are giving this child this medication that talks more into sexual intercourse, what are you saying to the future of this child? As much as you are protecting the child from possible illnesses that may hinder the same future that you are talking about, it is a bit of a dilemma but then at the end of the day it will morally and according to the values, it may not be correct or it may not feel correct. That is what discourages, but then at the end of the day you will go back and say you know what, we have this pandemic that we are dealing with.

1:62 ¶ 261 in Clinic A - FGD

PARTICIPANT 29: Yes and they are having sex anyway. So if I am denying her as a 15-year-old, I am saying go on, continue with the pandemic. So what is discouraging counts with me as a person or as an adult looking at this child and you know as much as PARTICIPANT 30 has mentioned, you are giving this child who still needs to be reminded about the homework, you are giving this child the responsibility of taking a pill each and every single day without the person who will say 'remember to take your pill'. This child in not sick, number 1, when a person is sick it is unlike those ones that are HIV positive he knows that you know what I am sick, if I do not take this medication

1:63 ¶ 263 in Clinic A - FGD

PARTICIPANT 29: I can… exactly so then you have this one who is just not sick. Whether she took it or she did not take it, the compliance is there definitely, it is something else. So given the responsibility to that 15-year-old who just is not so mature and then there are those that are generally sleeping around, those 15-year-olds who will sleep with 7 guys in 1 week, but the thing is when they come you do not see whether this one is that kind or is not that kind, so I give.

1:64 ¶ 265 in Clinic A - FGD

PARTICIPANT 287: Ja, unfortunately when you come back to when? I raised my hand and took an oath, unfortunately personal things they do not need to come, they do not matter even if I can see that basically for this person it may not necessarily benefit this person, but because this person came to me seeking for these, for whatever reason that is, the main thing that is important is to give information to that person. By the time that person leaves I would be the ones who is remaining behind and say PARTICIPANT 287, did you see now, did you see that? Okay fine, calm yourself down, this is life.

1:65 ¶ 267 in Clinic A - FGD

PARTICIPANT 29: Because you are sitting there and thinking this 15-year-old, it is not like that one who is actually positive, it is this one who is just negative and you are at day one at 15 years staring to interfere with this person's kidney, you are interfering with this person's liver, so for what, but then.

1:136 ¶ 12 – 16 in Clinic A - FGD

PARTICIPANT 28: My perception on PrEP is that we are giving these youngsters, can I say not an opportunity, I am looking for the proper word, a go-ahead to say go expose yourself to HIV.

INTERVIEWER: Okay, so you are saying with PrEP we are somehow pushing …

PARTICIPANT 28: We are encouraging them, yes.

INTERVIEWER: We are somehow encouraging them to go and practice unsafe sex. So why do you think PrEP does something like that to them?

PARTICIPANT 28: Because with… Okay I will talk you about my personal experience with my clients, because immediately when they come when you ask them are you going to start using condoms, they are like no sister I am not going continue using condoms because I am going to be protected from HIV and they never come back.

4:87 ¶ 278 in Clinic C - FGD

PARTICIPANT 40: What if she is twelve and she is sexually active?

4:88 ¶ 282 – 284 in Clinic C - FGD

PARTICIPANT 42 Because they are twelve, they take decisions, they can sign for themselves.

INTERVIEWER: And then participant two?

PARTICIPANT 40: Which drug are they going to use that time because the one that we are using, even with the other people, it is according to the weight and stuff, and then if she is twelve?

4:89 ¶ 287 – 293 in Clinic C - FGD

PARTICIPANT 43 I do not think that I would be confident to start a twelve-year-old on PREP.

INTERVIEWER: Yes.

PARTICIPANT 43 Yes, I do not think so.

INTERVIEWER: So, what would make you less confident?

PARTICIPANT 43 Their age.

INTERVIEWER: Why, what is bothering you about the age?

PARTICIPANT 43 The inside, I mean, mental readiness depending again. Like, before you even start, you need to prepare this person psychologically that she is ready. So, I do not think that a person, a child of twelve is psychologically ready to take such a drug for such a long time. Yes, so that is my take.

4:90 ¶ 295 in Clinic C - FGD

PARTICIPANT 40: That is where it leads to non-compliance. Because she is still young. I do not think that she or he understands what they are taking this medication for.

4:91 ¶ 299 in Clinic C - FGD

PARTICIPANT 40: At the moment I only know one drug that is given for PREP, and then, according to my understanding, that one is suitable for a certain weight. But a twelve-year-old-

4:92 ¶ 307 in Clinic C - FGD

PARTICIPANT 44 Really, I would say that I will also feel uncomfortable in that case, if it happens. Unfortunately, I never had that case of a twelve-year-old. But should it happen that maybe I have a twelve-year-old, who wants to be on PREP, I will not just issue it, irrespective of whether she qualifies, but I will take the patient to the social worker to find out what causes that child to want to indulge in sex. Because I will look at other factors, because it could be that maybe there is somebody that might be abusing her, giving her money or maybe it could be incest. So, in that case, definitely, I would refer it to the social worker, to intervene. I would not even encourage it at all, honestly speaking

4:93 ¶ 315 in Clinic C - FGD

PARTICIPANT 43 It depends again on, like I was saying, on the mental readiness of this child. So, if she is mentally ready and matured, I would dig deeper, like you say. And then send to somebody who is a professional to see why this child is like that, because obviously, there are expected behaviours, irrespective. Okay, other children mature faster than others, but there are expected behaviours around a certain age of children. So, if it is something alarming and I cannot resolve it, obviously, I will pass the buck.

4:94 ¶ 321 in Clinic C - FGD

PARTICIPANT 40: I would give to a twelve-year-old, after exploring all other things, but my problem, if there is a suitable medication for that child, I will give. My issue, at the moment, what I know that we are giving, I do not think it is suitable for a twelve-year-old.

4:95 ¶ 327 in Clinic C - FGD

PARTICIPANT 39Not even working the dosages or whatever, you must find out exactly what is happening, because it could be a form of incest or abuse by somebody who gives

money. You see, maybe they charge as an offense and somebody is taking advantage, it is true what you say.

4:96 ¶ 329 – 332 in Clinic C - FGD

PARTICIPANT 42 Remember the statistics are saying, our ten-year-olds are getting pregnant in schools. So, by the time they are twelve, they have already started there at ten. So, they are matured mentally, even if they are twelve.

PARTICIPANT 44 But there is no twelve-year-old who will tell you who is mature?

PARTICIPANT 42 By the time they are twelve, they have already been indulging in sex for how many years? More than two years already.

PARTICIPANT 44 But they are not-

○ Perceived barriers to PrEP uptake and retention among females: 2.2 Healthcare provider barriers. Inconducive attitudes

4 Quotations:

3:24 ¶ 205 – 207 in Clinic B - FGD

PARTICIPANT 34 Also have this tendency of saying young as you are why do you, it means you have started ...

PARTICIPANT 35 Started.

PARTICIPANT 37 Ja, with sexual .

4:77 ¶ 226 in Clinic C - FGD

Because the child would like, maybe to come in the afternoon, maybe even go to school, and they come in the evening where they will find me and my colleague, tired, and she wants PREP. Do you think we will offer that?

5:30 ¶ 115 in Clinic D - FGD

PARTICIPANT 48: The other thing that I am thinking of that [they hate, they stop in coming to collect the PrEP?. They come for the first time and was attended to very well and then when they come for the second time, the Sister is not there, they go from each room, they say the Sister working in this room is not here today and me I know nothing about PrEP, go to room number so and so. Then the child decides to go out and the other thing, the attitude that when I am telling that child that the Sister is not there and me myself I know nothing, the attitude that I portray to the child that makes the child never to

come back again to the clinic. So the attitude of the medical staff also affects the attendance.

5:53 ¶ 253 in Clinic D - FGD

PARTICIPANT 46: You can be friendly as an adult, as a granny like myself, you can be friendly to everybody, but what the other child sees [in you as such, the problem is they see my? hard face, so the problem is the young people, whenever they see your hard face, so the problem is now that they see the minute you come-

---

○ Perceived barriers to PrEP uptake and retention among females: 2.3 Information barriers. Inaccurate information dissemination regarding PrEP

14 Quotations:

1:3 ¶ 20 in Clinic A - FGD

PARTICIPANT 29: so from my personal experience as well, I have experienced a number of clients who would come and get PrEP. I do not know it seems like that at times they take PrEP as PEP because they cannot take PrEP for that month and then it does not matter how thorough you explain to them they just happen to not come back. Most of the clients do not come back for PrEP, they take it for a month and then they are sorted because at most they come because she had sex or he had sex the previous week and then he is coming in the 72 hours has lapsed for PEP then we suggest PrEP, she takes PrEP and then it will be for a month and then done.

1:4 ¶ 22 in Clinic A - FGD

PARTICIPANT 287: On my view I would say the reason why most of them after they have taken PrEP they do not come back necessarily, as I would concur with PARTICIPANT 29, with that they come as… thinking that after I have taken PrEP on that point where I have done maybe that moment of infidelity or maybe that point of being careless, then that means I am okay,

1:6 ¶ 24 in Clinic A - FGD

Even if you can explain because you can go as far as Cape Town and come back, trying to illustrate that the side effects they come with each and every medicine or any regime whatsoever but it all boils down to the body getting adjusted and accepting what I am taking right now and even if you can go as far as also explaining that after this medicine or after the PrEP is now used to your body, you are definitely not going to even actually know that it is even there. It is just that it is only you in your mind that okay, I am used to it now. So how about now when you are putting it onto a client okay, fine, you have left

this PrEP now for this moment, have you also left the No, it is not possible, it is part of life, but then again, what is going to happen now so when you have contracted something that is going to make you now stay on ARVs for life whereas you had a chance of staying on something that can protect you?

1:7 ¶ 30 in Clinic A - FGD

PARTICIPANT 287: They continue having sex despite the fact of knowing that I have decided to stop taking PrEP because of the side effects that even though Sister did explain that there are side effects even though those side effects do not last long, but you know.

1:48 ¶ 197 – 198 in Clinic A - FGD

PARTICIPANT 28: a lot of initiations and they never come back.

PARTICIPANT 287: They never because most of then they will think they are only taking it for 7 days and then after 7 days I am done.

1:49 ¶ 201 – 202 in Clinic A - FGD

PARTICIPANT 29: some perceive it as PEP.

PARTICIPANT 287: Ja you understand, because something happened the day before yesterday, now-

1:50 ¶ 203 in Clinic A - FGD

PARTICIPANT 29: And some they are thinking it is just for general STIs.

1:66 ¶ 269 in Clinic A - FGD

PARTICIPANT 287: And just to add on something else that is also discouraging when it comes to PrEP, you can be looking at what was the initial point of initiating this person or maybe what was the reason behind initiating this person and it comes to compliance and this person does not pitch up and then this person decides to come back after 6 months down the line and has been missing, what has been happening in these 6 months and then you are looking at the stationery. It is gap, it is gap, it is gap, she last took the initiation, 6 months down the line she never came back. She is coming back afterwards so it all boils down to [the meaning and understanding of adherence and also the client needs to stipulate for how long are you planning to stay on these because now you say that you need it, how long are you planning to stay on it.

1:131 ¶ 351 in Clinic A - FGD

PARTICIPANT 29: Generally it is the knowledge of our clients, that also plays a big role, how literate our patients are and how knowledgeable they are in terms of HIV, in terms of medication. It goes deeper, there are some clients who would come, you talk to the clients and through the conversation you, even if he says no to PrEP, he says no because he understands what PrEP is. He is not saying no because I do not want many pills, he

understands what PrEP is and there is this client who will just say It does not matter how deep you go in explaining what PrEP is, what it is for and it is a client that this male client who has confessed to having multiple partners, who has confessed to not being circumcised but you are offering PrEP, he still says no and he confirms as well that he is coming several times for STIs, he does not like using condoms but still says no to PrEP.

3:37 ¶ 343 in Clinic B - FGD

PARTICIPANT 32: If you're taking PrEP that automatically means you are not using condoms.Have unprotected sex.

3:52 ¶ 397 in Clinic B - FGD

PARTICIPANT 33 The females it will be in terms of their young age. Because the young girls most of them are problematic issues, even if they can get the information, purebred they are misled even if she has the right information, she will be misled until she even doubts the information she is having.

4:4 ¶ 17 in Clinic C - FGD

PARTICIPANT 41: You know, I think the way that PREP was introduced, it was not well introduced. Because our teenagers think that PREP will prevent them from getting HIV, hence they are taking it. But now, along the line, they do get HIV positive. So, according to them, I think they think that when they take PREP, they are not going to be HIV positive. Hence, they take PREP and do not use condoms. So, they become infected. You know, again that, because we do not get retention of these young girls, it is because they now fall pregnant, I mean, they get HIV along the line. Three or four of those are now HIV positive, because they were not using protection. So, they were thinking, if they are taking these tablets, they are not going to get HIV. But now, they do get HIV, and then why we have this much rate of getting them retained, it is because now if I see that my friend, we were both on PREP, now she is HIV positive. So, this one also leaves PREP, so as to say, I thought I am not going to be HIV positive, but now my friend got HIV, you see.

4:63 ¶ 15 in Clinic C - FGD

PARTICIPANT 40: Yes, I agree it is important, but I think there are challenges there, as much as I think it is important. I think there are many challenges that must be dealt with. So, there must be consistency so that we must also achieve what we want to achieve. Because we might talk PREP, PREP is important, but these kids, do they understand how important it is? Because if they do not understand, at the end of the day, they will not comply.

5:51 ¶ 49 in Clinic D - FGD

PARTICIPANT 49: Yes, most of our young people, they are taking this particular treatment today and then tomorrow they [will not actually come back or they do not need treatment and? continue with it, because they have actually discovered after taking it that

these are actually ARV's of which that information they never actually have been provided with on the day when they were actually initiated on this particularly treatment, so after discovering that this is like I mean the ARV that I am taking, the person thinks, it means they actually say that I am HIV positive now. So it becomes a problem.

○ Perceived barriers to PrEP uptake and retention among females: 2.3 Information barriers. Ineffective information dissemination regarding PrEP

7 Quotations:

3:8 ¶ 69 in Clinic B - FGD

PARTICIPANT 33: And always the problem to take this, it's a lack of information, the importance of this and how long should they take it. And the benefit of it.

3:25 ¶ 214 – 215 in Clinic B - FGD

PARTICIPANT 35 For PrEP, but what is including is that they are saying it's not well advertised. Because others will say you know I didn't know about the PrEP.

So I my friend but they saw its not well advertised, meaning I don't know whether its social media or what.

3:26 ¶ 221 in Clinic B - FGD

PARTICIPANT 36 Ja, its very, its correct because when you go into social media, you hardly see adverts about it. So I also emphasise on that, to say it's not really, we don't reach the target market for PrEP.

4:16 ¶ 29 in Clinic C - FGD

PARTICIPANT 43 Yes, PREP is good, it is a good approach. But, in as much, I do not think that the youth, or the young people are informed, well informed about it. Even the healthcare professionals, I do not think that they are well informed about it. It is only a minimum amount of people that know about it. Actually, it is not well marketed, if I may say. So, maybe that is why our statistics are very low because of that.

4:33 ¶ 53 in Clinic C - FGD

PARTICIPANT 39Like, participant five said, PREP is not well marketed. We do not see much of it on TV. How does it work, like ordinary ARV's, when it is started?

4:43 ¶ 138 in Clinic C - FGD

PARTICIPANT 39I think that the baseline health education was not given about the frequency of bloods and why they have to take blood for.

6:36 ¶ 171 in Clinic E - FGD

PARTICIPANT 53: I think even chronic, the information is lacking, I do not think the patients are getting more information.

○ Perceived barriers to PrEP uptake and retention among females: 2.4 PrEP related barriers. PrEP folllow up/monitoring burden

27 Quotations:

1:100 ¶ 400 – 402 in Clinic A - FGD

PARTICIPANT 287: Yes, but then again I go back to your point, then when do you monitor HIV every 3 months? Because if you are giving them that collection point somewhere, [unless that is what you are? doing as in now, when do you do the monitoring of [the HIV?

PARTICIPANT 29: There are testing points all over so they can go test... okay, but going there, there is no one controlling even their own. If there was someone controlling they would produce the test results and then you…

PARTICIPANT 28: Remember [if you can take? you must meet specific criteria or conditions so with PrEP [you have to be HIV? somebody has to supervise it. So it [won't work with? PrEP.

1:118 ¶ 353 in Clinic A - FGD

PARTICIPANT 287: To what I have encountered, I knew at the beginning of starting or initiation we need to take those bloods, how often are we out of equipment.

1:119 ¶ 357 in Clinic A - FGD

PARTICIPANT 287: I as a professional nurse I had to scramble around the clinics in my own time, my own transportation to get the bottles for my clients or my client base and even actually for the [whole thing for? whoever are coming to get those. Why do I have to hassle so much?

1:121 ¶ 318 in Clinic A - FGD

PARTICIPANT 30: I concur with the other participants that coming for clinic visits every other 3 months could be a challenge

1:123 ¶ 320 in Clinic A - FGD

PARTICIPANT 29: Waiting period as well, it still comes back, waiting period generally in the clinic, I am not sick. I have to go there wait in that long queue just to get the pills, I am no longer going there.

1:124 ¶ 316 in Clinic A - FGD

PARTICIPANT 29: where I am employed and where I am working this coming every 3 months here is not working for me. I am going to lose my job so I am not sick, why should I take something that might make me end up losing my job. So I do not need to take it

1:125 ¶ 322 in Clinic A - FGD

PARTICIPANT 287: And the other thing that even if maybe he is already in the queue outside where you as the clinician you are inside and you are finding this, I would say … is it correct for me to say problematic kind of pace and you cannot say because this one is inside already and you were discussing something that you can see clearly this person is not understanding. You cannot just say ai, ai, ai your time is up, you cannot do that, it is unethical. You need to make sure that this person when he leaves the room is satisfied with the information that is given to the person. What about the one that waiting outside who has been waiting there and I not sick as we are saying, he is not sick he is just coming for this pill. By the time you open the door, next one please, that person is gone.

1:126 ¶ 324 in Clinic A - FGD

PARTICIPANT 287: Not about the long queue, there is someone who is inside they are not patient amongst each other.

1:127 ¶ 326 in Clinic A - FGD

PARTICIPANT 287: The one that was inside maybe the problem was bigger than what was anticipated thinking that maybe she is just going to come in and then take the pill and then walk out. It is not just as simple as that because mind you when nursing someone or you are taking care of someone it is not just a pickup and go. You also, all the aspects that the elements of a human being they are being catered for before you can let go of that person and as a result the one who is outside-

1:128 ¶ 334 – 335 in Clinic A - FGD

PARTICIPANT 287: And the very same taking or monitoring of blood and you will find that you will tell the client, you know that we must take blood at the initiation and you follow it 7 months later, ja as you go forward as long as the client is on therapy. And it is only 1 room that is taking blood in whatever facility [that is maybe just randomly?

I am not sick and the queues that are there and that pain.

1:129 ¶ 337 in Clinic A - FGD

PARTICIPANT 287: Initiations and 7 months later you monitor the kidneys and then every year as long as you are on it, on the same day you started you go back and you find that person maybe by that day, maybe I was somehow dehydrated, the veins were quite difficult to find, that pokng, poking, I cannot …

1:130 ¶ 343 in Clinic A - FGD

PARTICIPANT 287: Yes even if you do explain the fact that the reason that is behind me testing you every 3 months when you are coming is that also, it is not because I do not trust you but it is also just to ensure that the medication that I did give you, you are taking it accordingly and you have not contracted anything for us to maintain the eligibility to continue…to others it is a story

3:12 ¶ 95 in Clinic B - FGD

PARTICIPANT 34 I think one of the side effects besides that, TDF that she spoke about, having them to come to the clinic every month; I don't know how you issue it.

3:17 ¶ 149 – 150 in Clinic B - FGD

PARTICIPANT 37 They not going to they won't come because they're students. They'll be telling, especially the, most of them they are matriculants.

Grade 11, Grade 12, they've got, doing extra classes they're coming late after, usually they come after 4 at school. So they don't have enough time. They're always rushing. And maybe that could lead to poor compliance.

3:58 ¶ 430 in Clinic B - FGD

PARTICIPANT 32: It will be a challenge because me as a 165 year old I don't want to go ... chronic medication that takes forever and now I'm just wanting PrEP, I'm not sick, for me. .

3:66 ¶ 483 in Clinic B - FGD

PARTICIPANT 33 I think, another thing it was space that time and it was mixed with chronic so again it was waiting time, so they would run to our, if they see they would run straight to our kids side and then they will go home. So we were missing them, but we had so many kids there at that time.

4:5 ¶ 19 in Clinic C - FGD

PARTICIPANT 41: Yes, and again, the ones that took it, not knowing that they might be HIV positive, they come back and say, but why, each time do I have to come back to do the testing? So, that makes them aware that you might be HIV positive at any stage during the taking of PREP. Then they say, but if you are doing family planning, when you come back for your date, your appointment, they do not test you for pregnancy. So, that means

family planning is reliable. So, this is not, because each time you come back, they have to do the HTC on you.

4:10 ¶ 448 in Clinic C - FGD

PARTICIPANT 42 I am just thinking about the testing. I agree with all of it, to say, the testing should stay for three months. Because if you are going to leave it for longer, they become pregnant, they become HIV positive, by the time you realise, the child is six months pregnant. So-

4:49 ¶ 416 in Clinic C - FGD

PARTICIPANT 41: Because again, if you are saying we are going to test them once or twice a year, we are going to miss out. We are going to miss them, to say that they are now positive. I think the testing part, there is nothing we can do about it.

4:50 ¶ 409 in Clinic C - FGD

PARTICIPANT 41: Because now they testing, is it monthly? When do they come back, each visit? They come every month for three times-

4:61 ¶ 208 in Clinic C - FGD

PARTICIPANT 43 Yes, it is like a burden again, to say that people must move from their catchment area to come to a certain facility, where you carry all the people that are staying there and beyond, like the ones that are coming from other areas. It kind of, again, brings, the defaulter rate becomes high. Because if that person does not even have money to come to that facility, she would not pitch and because of, like I said, the relationship that they had, a person would even be afraid to come maybe a week after, to say, I did not have money the previous week. More especially because they have knowledge that it is not like they are sick. It is for prevention.

4:65 ¶ 134 in Clinic C - FGD

PARTICIPANT 39He came in and I told him that today we have to take bloods. He said, what? For what? I did take bloods, and that lady was stabbing me, and why should I take bloods? I had to explain that we have to check his kidneys. I explain the programme, how often. He said that he does not know, he is scared of needles and he did not know that we would have to take bloods, and he did not understand why.

4:70 ¶ 212 in Clinic C - FGD

PARTICIPANT 43 Yes, because like you said when we started, the facilities that you have mentioned are CHC and then from there, there are also my core clinics that are happening around that CHC. So, the CHC are just not next to the clinic. So, a person who can walk into a facility, a clinic, has to have money to go to that big one, the CHC. So, they would come maybe once or twice, and then from there, because they do not see it as important,

like they are sick or anything, it is just for preventative measures. The patient will not even come.

4:75 ¶ 226 in Clinic C - FGD

And the number of times that they need to go to the facility for their bloods and everything again, it is also a problem

5:40 ¶ 213 in Clinic D - FGD

PARTICIPANT 47: What I have learnt about young people, they want a drive-through fast-food kind of service. If you make them wait they are definitely not going to come back because remember they are not sick so they hate this waiting. So only if it can be some kind of education this window, tablet at this window, off you go. You promise them that it is not going to be more than an hour, I am telling you they will come, but the next chair, the next, you move they will not come.

6:31 ¶ 106 in Clinic E - FGD

PARTICIPANT 55: that needs to be done take blood, maybe that might be the reason why they are not coming back.

6:32 ¶ 108 in Clinic E - FGD

PARTICIPANT 54: So if even if we did have a patient and the patient was also [agreeing that okay fine? I will take PrEP and then you will be sitting with a patient who like okay let me take blood for you, they are like ha, you are supposed to take blood, I do not want it anymore.

○ Perceived barriers to PrEP uptake and retention among females: 2.4 PrEP related barriers. PrEP pill burden

11 Quotations:

1:10 ¶ 46 in Clinic A - FGD

PARTICIPANT 30: but I am also of the perception that we are saying that adolescents who are around 15 and above can also access PrEP but I am looking at the level of maturity for someone who will be taking treatment every month and also looking at an aspect of if you are going to be taking medication for a long time to prevent, there will be fatigue. So my perception is most probably the reason why we are also not retaining is if we are saying that this person has to take it for a longer period what are we saying to treatment fatigue even if it is for prevention?

1:11 ¶ 48 in Clinic A - FGD

PARTICIPANT 287: What I can maybe add into what she is saying, or maybe just a little bit be [aware of it?, maybe that a client who may say I will be on this treatment for the very longest of time, how many of us in this room was like constantly in a relationship and having sex every time. There are times whereby you are in a relationship and when you are in a relationship obviously, sex it is part of the game but there are times whereby you do not want to see that thing next to you. You understand?

1:28 ¶ 112 in Clinic A - FGD

PARTICIPANT 29: I have reservations on it. Reservations, I am going to go back and look into side effects, look into having to drink a pill daily, look into so many reasons that we get normally from patients who would say I am not going to take PrEP because of 1, 2, 3. So if we are able to turn around those reasons that make people to not want PrEP, like you say no I cannot because I cannot be taking a pill everyday or the side effects of the medication.

1:54 ¶ 241 – 243 in Clinic A - FGD

PARTICIPANT 30: Discourage me would be, I am of the notion that a 15-year-old to an 18 year-old is still an adolescent yes, but a minor.

INTERVIEWER: So because they are minors you would not give them?

PARTICIPANT 30: No, I would give them, yes it is just … I would give them because if they are sexually active and they are not HIV Positive and they are eligible for PrEP then we would give them but it is a little discouraging because this person is a minor who is still… I am just looking at the maturity level of a 15/16 year old who is going to be taking this medication every day at the same time at night, that would discourage me,

3:4 ¶ 28 in Clinic B - FGD

PARTICIPANT 32: So some of them might have the mentality of why am I taking medication when I know that I'm HIV negative.

3:5 ¶ 34 in Clinic B - FGD

PARTICIPANT 32: So some of them might have the question of why am I taking ARVs when I know that I'm HIV negative.

3:22 ¶ 178 in Clinic B - FGD

PARTICIPANT 36 So having to take a drug for lifetime, it’s a difficult one.

4:11 ¶ 142 in Clinic C - FGD

PARTICIPANT 43 The pill burden, no one expects to, it is like you are attending to a chronic person for something that you do not have. So, it is a problem, especially if maybe

the patient was not well informed in the beginning. Because if you take Panado, you expect to be healed by that moment. But if you have to take tablets for a long time, obviously along the way you are going to default, and sometimes because you know you do not even have what you are preventing, I think the mindset would just what am I preventing, actually, because I do not have i

4:17 ¶ 33 in Clinic C - FGD

was only maybe one or two who stopped PREP, because they said, this thing of taking a tablet daily is boring, because at times we are with our boyfriend or girlfriend, then I see that I have left my stuff at home. And then, they will tell you, things happen.

4:44 ¶ 144 in Clinic C - FGD

PARTICIPANT 43 Especially if they have to take it on a daily basis. It goes back to that, if you take something on a daily basis..

6:5 ¶ 15 in Clinic E - FGD

PARTICIPANT 54: Okay, so for… What I have heard is they do know the dangers of sleeping with somebody without, right, but then again they also do not like pills, so taking pills every day is a bit difficult for most of them. So yes, basically they will not really take PrEP because of the reason that it has to be taken every day, because they will ask, do I have to take it every day or do I have to take it whenever I am sleeping with a person on that time or after. So for the fact that they have to take it every single day for it to be effective it kind of also is a problem.

○ Perceived barriers to PrEP uptake and retention among females: 2.4 PrEP related barriers. PrEP side effects

7 Quotations:

1:5 ¶ 22 in Clinic A - FGD

PARTICIPANT 28: but not bearing in mind that to some of them they also have this thing that they do not really understand that why do we have to take something that is meant to protect us when it comes with side effects because some of them the moment they encounter any of the side effects that comes with PrEP, that person automatically just stops and when that person stops, even if you can come back and you see that person for maybe family planning or anything else or maybe the very STI, you ask but according to the data, I have you and I remember you for that matter that you are one of the clients that I did initiate you on PrEP but why did you now stop? Sister you did not tell me that this thing is coming with this thing, I was not coping.

3:11 ¶ 87 in Clinic B - FGD

PARTICIPANT 35 When we, maybe give them information about the effect, the side effect of ….So always they come and ask to say you know I wonder, you know with the results. Or what will happen to my kidneys, I say no we are having kidneys every time when they come to the ARV but that is the other thing that they are concerned about.

3:20 ¶ 168 in Clinic B - FGD

PARTICIPANT 31: They only talk about the headaches. And some of them tell you they're gaining weight. Nausea. Nausea, mmm.

3:21 ¶ 172 in Clinic B - FGD

PARTICIPANT 36 They usually stop it when they experience that they stop them

4:6 ¶ 27 in Clinic C - FGD

PARTICIPANT 42 Yes, PREP is very important. I also stand with participant two, to say there are so many challenges. With me, I am worried about the kidneys of these children. Imagine from the age of twelve, a child is taking that treatment of PREP, and by the time they reach sixty, where will their kidneys be? How will their kidney function be? So, that is what I am worried about most.

4:55 ¶ 384 in Clinic C - FGD

PARTICIPANT 40: I think if it is what participant four has said. Like, we have concerns about the medication that we are giving have a lot of effects on the kidneys and stuff. So, we have that worry. Maybe we do not market it that much, like a sixteen-year-old comes and then I am like, I am giving this child from now on until, then those kidneys will be damaged by then. Maybe that is what-

6:28 ¶ 96 in Clinic E - FGD

PARTICIPANT 54: Yes so I feel like most of the time it is just side effects, so immediately when they are experiencing side effects they just leave the PrEP immediately, like some other ones are even saying they felt like they were dying, so obviously if somebody is feeling like they are dying they are not about to continue taking it. Yes.

○ Perceived barriers to PrEP uptake and retention among females: 2.5 Youth related barriers. Limited awareness and don't care attitude

8 Quotations:

1:132 ¶ 456 in Clinic A - FGD

PARTICIPANT 287: I believe there is something that we may be leaving out a bit. We are dealing with clients that some of them they fail to be transparent and truthful to the extent that even the very information that the person is giving, it is not truthful. Even if you can try because right now we are trying to reach out by doing the reminders, making the phone calls, when was your last visit and when would you be able to come back for your follow-up and so forth, you will find that the number does not exist and then it boils down to the fact that, that means this person the trust from the beginning was something else in as much as I was unable to give you, who am I.

3:49 ¶ 383 in Clinic B - FGD

PARTICIPANT 31: Its like that, most of the time they usually come to the clinic for family planning, they are more worried about pregnancy than HIV. So we recruit them from the family planning side and try and give them information about PrEP, so make them aware. If they are sexually active and they are worried about being pregnant, they should also consider that there is a possibility that they could become HIV positive.

3:50 ¶ 391 in Clinic B - FGD

PARTICIPANT 37 I think with females they are in denial because they even don't know when they have discharge. They will just say it's a normal discharge, but when we go deep you will see it's an STI. So we deal with these kids like I don't really.

3:51 ¶ 393 in Clinic B - FGD

PARTICIPANT 37 I think girls they tell themselves its normal for a girl to have a discharge but they don't know the different discharges. They say it is okay and so that's normal. But ja I was thinking its normal. So guys they know that once it comes, you are dying.

3:69 ¶ 349 in Clinic B - FGD

PARTICIPANT 37 The kids they are not staying with them moms, they can take their medication in the morning and go gallivanting the whole day with their partners. I don't think that can stop them.

4:47 ¶ 162 in Clinic C - FGD

PARTICIPANT 39I totally agree, like really, let us be realistic here, even a course of antibiotics, I think it depends also on maturity. Those that are twenty-three, twenty-four, they are a little bit more mature, they know what they want, others are at tertiary, their compliance can be good. But our eighteen, seventeen-year-olds, it is like they thought, like participant three said, they thought that this was a one-tablet fix. It fixes everything, no condom, nothing. You just take it when you are going to your boyfriend to sleep over and then that is it, you do not have to take it again. If the boyfriend stays in Durban, you just take it when you go over there for the weekend. When you come back to Joburg, you do

not take it anymore. And that sleepover, there is no condom. They thought it was something that was going to fix all their problems. They take that tablet when they feel like taking it, then it will solve their problems then.

5:55 ¶ 19 in Clinic D - FGD

PARTICIPANT 51: Okay PrEP neh… it is, okay, on both sides, it is good and it is also bad. It is good in a way what participant 2 and 1 said and then it is bad because I feel it promotes the young ones to be reckless and just have multiple sex partners because they know it can prevent themselves from HIV. So it is bad in that way. So it is good and bad.

6:41 ¶ 113 in Clinic E - FGD

PARTICIPANT 54: whilst with the young ones, like with the status, they do not know the status of their partner or anything like that, so at the end of the day they are not about to take PrEP because they feel like what is the point, especially because of taking it every single day.

○ Perceived barriers to PrEP uptake and retention among females: 2.6 Social barriers. Parent related barriers

12 Quotations:

1:30 ¶ 118 in Clinic A - FGD

PARTICIPANT 28: Disadvantages according to my patients, one of the disadvantages, they need to hide this medication. You must remember my clients are from 15 to 24, every time I try to engage with them why do not you come back, oh sister, but I need to hide this, what am I going to tell my mom? I am on this ARV because we need to be honest that they are taking ARVs, we cannot say we are taking a pill. These people they will go and Google and they will get frustrated, they will come and shout, but you did not tell me this was an ARV. So one of the disadvantages if you live with your parents and they see it. Immediately with lack of information they will think you are already HIV positive.

1:33 ¶ 120 in Clinic A - FGD

PARTICIPANT 30: Also we are talking about adolescents who are under the care of their parents, firstly this child is already alluding to the fact that they are having sex which may be hidden to their parents already and there is not much of knowledge and support on PrEP especially if it is a 15/16/17 year old who is still under the care of a guardian or parent.

3:9 ¶ 73 in Clinic B - FGD

PARTICIPANT 36 Ja, I think also we, we tend to look at the young girls. Not knowing that they come from a family. So you initiate the young girl coming in and don't involve the parents. And when they get home with that container automatically they are taking a drug which is ARV. So it exposes them to saying yes you are having sex number 1. And you are taking the drug. Number 2, why are you giving my child ARVs without consulting me. You know so we, with that also it brings that attitude to say eish if I take this what will my mother say. If I involve my mother, so wena you're having sex. So that's the point of attitude. That you have to desensitise best.

3:10 ¶ 77 in Clinic B - FGD

PARTICIPANT 37 Ja, that's another challenge that we're having because parents tell their kids to stop it because it's an ARV. So they will come back and say my mom said I must stop this, even with the information is there. And even the child can try to explain to Mom it's not, it's not that I'm positive. It's the prevention, they that but they still don't want to understand that.

4:25 ¶ 86 in Clinic C - FGD

PARTCIPANT 39: Everybody must know, because if these kids are, I know, like participant six said, they are scared to talk about sex. They do not want their parents to know that they are sexually active. I know that there is a lot around this thing.

4:35 ¶ 84 in Clinic C - FGD

PARTCIPANT 40: Because now, at home, if I am not well informed about PREP, and then my sixteen-year-old comes with a bottle of whatever that I do not know, and then that is how it comes to stigmatisation.

5:11 ¶ 59 in Clinic D - FGD

PARTICIPANT 51: And another thing because, when they get home they realise ARV's and some get shouted by their parents, because some of the parents they do not know they came to the clinic and they took PrEP. So obviously if a parent sees that thing, they will start calling me, questioning me? , asking me why is my child taking ARV's. Now I need to explain no they are not ARV's, it is PrEP.

5:13 ¶ 61 in Clinic D - FGD

PARTICIPANT 48: The other thing, it gives the kids another stigma from the parents that, that means now you are sexually active, so that is why you are taking these tablets

5:14 ¶ 61 in Clinic D - FGD

PARTICIPANT 48: The parents only know the transmission of HIV as sex. They will never think of the injuries like participant 4 talked about, being involved in an accident or whatever. They will never even think of in cases of rape. The mere fact that my daughter is taking that, that means she is sexually active and maybe being promiscuous in that action

5:28 ¶ 101 in Clinic D - FGD

PARTICIPANT 47: What I have seen or what I have heard from them, some of them it is lack of support from parents because the other one came and said, Mummy said and then the child had to leave PrEP. The Mummy kept saying so the child was like Sister I am leaving this.

6:7 ¶ 19 in Clinic E - FGD

PRATICIPANT 3: Yes, they feel every day like they are taking ... and another thing, I think stigma and disclosure. We once had parent who came here complaining we gave her child ARV, she is HIV positive, she knows ARV's and everything, so the child came back home, medication, she found the medication, she brought the medication back. So this girl I am taking this, so this it means you are sexually active and you are not using protection, this girl decided to hide the medication, after disclosure and, if you are taking this treatment you are HIV positive.

6:8 ¶ 21 in Clinic E - FGD

PARTICIPANT 53: It is why we will need to teach them, I do not know if to teach or counselling for those kids, who are not too scared to tell their parents, as she is saying the Participant number 3, one day there was a patient ... or there was a parent, she was a single mom, she was so furious because she is saying we are teaching their kids to engage to the sexual while they are not and then she said her child she is so quiet, she is from Botswana, so what we are teaching her, she is going to open the case for us.

○ Perceived barriers to PrEP uptake and retention among females: 2.6 Social barriers. Partner related barriers

5 Quotations:

1:31 ¶ 118 in Clinic A - FGD

PARTCIPANT 28: Having to explain to your partner that you are taking this medication, already it is going to cause disharmony in the relationship if the partner does not understand.

1:32 ¶ 120 in Clinic A - FGD

PARTICIPANT 30: I do concur with what she is saying, there is a huge stigma that would be attached to, also if the partner finds you with the ARV themselves that oh is it that you think that I am going to expose you to HIV also.

1:89 ¶ 330 in Clinic A - FGD

PARTICIPANT 29: Oh no, just going back to the that you have pointed earlier on in what may discourage the patients, same as having to explain to the parents I am on PrEP, to your partner I am on PrEP, those can be discouraging that okay I am taking this now I have to explain to people why I am taking this. So there is quite a number of things that may be discouraging.

3:38 ¶ 347 in Clinic B - FGD

PARTICIPANT 38 I think it will because if I want to take it, I will make sure that the partner is not around. I will be able to take it, but if he is around there is no way that I will be able to get that, that right. Then automatically that will lead to me not taking the medication.

4:76 ¶ 226 in Clinic C - FGD

Because now you cannot be giving your child, they do not know. If you do not know, me as a parent, I do not know that my child is taking PREP, because they hide these things. The child would be saying, I am going to the clinic month one. Month four the child wants to go to the clinic. Obviously, it is suspicious, what is my child going to do there? Because of the level of education, again, with the tablets they are taking, remember they are also young, irrespective that they are used for PREP's. Other parents, they are even wise, they even read that and Google it. When they see that their child is taking ARV's, obviously, it is a stigma from even the house, that when I am taking ARV's, I will not tell you.

○ Perceived barriers to PrEP uptake and retention among females: 2.6 Social barriers. Stigmatisation

8 Quotations:

1:34 ¶ 130 in Clinic A - FGD

PARTICIPANT 287: All right, bear in mind that there are other therapies that we are using like managing HIV that is integrated into the very PrEP. Like if you are taking itself and you are using with the other additional drug, you understand, when you are treating according to, what is it, what regimen for that particular client. The moment they see that bottle of PrEP they will think that other one who is number 3 is somewhere … That is why you will find some of the clients that are with decant, they leave the containers in the facility.

1:35 ¶ 140 in Clinic A - FGD

PARTICIPANT 287: And they put it in another container or maybe in a plastic or maybe in whatever, the very same way an HIV positive who is on ARV does sometimes to hide this thing that is following him or her wherever she goes. So they do the very same thing.

3:6 ¶ 65 in Clinic B - FGD

PARTICIPANT 31: Okay the impact could very huge. Remember the stigma around HIV.When people think that you are taking, they see PrEP, they think that you are taking ARV, so people run away from taking PrEP because of the stigma towards the PrEP.

3:7 ¶ 69 in Clinic B - FGD

PARTICIPANT 33 Ja, I wanted to add the very same, of the issue of the stigma and since we are having our young girls, as the high risk for HIV.

4:34 ¶ 55 in Clinic C - FGD

PARTICIPANT 39Because when I put my bottle there for PREP and then somebody else who is taking real ARV's, they do not see any difference, they are all ARV's, it means that I am already positive to other people. So, you will find that no, I do not want to be associated with people who are taking ARV's.

4:36 ¶ 84 in Clinic C - FGD

PARTICIPANT 40: Again, what are we trying to do there? It means we are encouraging stigmatising HIV. Why are we supposed to stigmatise PREP? What is wrong with PREP? Because PREP to me is like family planning. They are HIV negative, they are just preventing. That is why I am saying, this youth, they need a lot of education. Maybe not the youth only, even the community at large.

5:9 ¶ 51 in Clinic D - FGD

PARTICIPANT 47: Because a stigma still exist with HIV, I think that is the reason they stop and they are never coming back because the stigma with people who are taking ARV's and their child is told you are also taking ARV's and they just stop.

6:29 ¶ 98 – 100 in Clinic E - FGD

PARTICIPANT 53: Even the what you call, peer pressure, is contributing, yes, because they laugh at each other due to lack of information.

INTERVIEWER: So you are saying another contributing factor, not only side effects, but peer pressure do contribute, saying some who might laugh, who are laugh at the other-

PARTICIPANT 53: They are saying there is stigma, there is still a stigma outside.

○ Perceived benefits of using PrEP: 1.1 Young females control over sexual health. Prevention in case of risky sexual practices

7 Quotations:

1:24 ¶ 98 in Clinic A - FGD

PARTICIPANT 29: Ja, I can add on PARTICIPANT 287 … I can add on what PARTICIPANT 30 is saying of which basically I think is what is being perceived as correct and of which is correct, but more than anything else bearing in mind that as a youngster you cannot be committed and be in a relationship that is fixed from that young age of 15. You are still exploring. I am still seeing Jonas Sono, I am still seeing Thomas, I am still seeing Tshepo. You understand, like I am in that you know, especially in the university life or maybe that high school life where you get to explore, you are there maybe for whatever and then that person maybe decides to disappoint you for whatever reason and then you are out of that relationship. You already made a plan of the sex contact with that person and then you go onto number 2 maybe after whatever you have healed, what are you taking to number 2. So for you to stay on PrEP basically while you are still searching for whatever because you cannot say youngsters they can, I can stick to this one, I am waiting for the right person to come along and so they definitely are, they are not actually, they are not sticking to, keeping to one person.

1:25 ¶ 100 in Clinic A - FGD

PARTICIPANT 29: They are just almost and more overly so with alcohol being part of the thing that some of them, they will wake up not having known who I slept with yesterday, but if PrEP is part of you definitely when that happens you wake up in the morning, you do not have to wake up with a status.

1:76 ¶ 286 in Clinic A - FGD

PARTICIPANT 29: There are clients who come and say the weekend that I am going to is very rough

1:77 ¶ 278 in Clinic A - FGD

PARTICIPANT 287: The groving and the reason behind me taking the PrEP because wanted to explore and …

4:59 ¶ 168 in Clinic C - FGD

PARTICIPANT 39it is a Monday, I will tell you that it cannot work today. To start with, you must make time, which is comfortable for you, which is convenient for you, maybe 6:00, 7:00, 8:00, a time which is convenient and maybe after a week it is then that it will be effective and you continue for a while. As long as you indulge in sex, you explain to them, as long as you indulge, you must take it. But, when you feel that maybe after six months you no longer have a relationship, you feel like it is useless, you can stop it.

5:5 ¶ 37 in Clinic D - FGD

PARTICIPANT 48: It is a good thing. I agree with all the other participants, but I agree more with participant number 6 that it also on the negative side, the kids just like to experiment then they just go around. The other thing is that our youth now these days they are too much into alcohol and then they will just be like and then at the same time on the positive side, especially they are gallivanting every now and again and they end up being raped so that PrEP is going to going to be helping them in cases of rape.

6:3 ¶ 9 in Clinic E - FGD

PARTICIPANT 55: I am agreeing with them, it is important to take PrEP, especially on young teenagers, because most of our young teenage girls some of them they are, some of them they are still virgins, maybe if you meet a boy, you do not know if that boy was, some of them from the year 2000, they are born with HIV, you just meet him, you do not know his status, you sleep together, you get infected, because some of our young girls they are very ignorant and she is still a virgin, but they just sleep without out using condoms. So if they are not on PrEP, they are not protected from contracting HIV, although it is not 100%, but still, yes-

---

○ Perceived benefits of using PrEP: 1.1 Young females control over sexual health. Prevention in cases of untrustworthy or HIV positive partner

8 Quotations:

1:21 ¶ 84 in Clinic A - FGD

PARTICIPANT 30: Yes as long as you know that there are moments whereby maybe the condom can be burst or maybe they are not using the condom or maybe I know because we are also focusing on clients who are really having partners who are HIV positive whereby maybe at the point that client who is positive is married, [or rather their partner is? and even may be on ARVs per se, but has not suppressed at the time so you know that there cannot be being in a marriage whereby it is just condom, condom everyday, condom everyday. They also get that thing of saying going to use this thing forever now, you understand? So you are just making to keep also their household intact knowing that as long as you are not contracting any other thing that we do not know about from this household like other STI’s. At least when you are on your ARVs and you are staying on your ARVs suppressing the virus and on my part I am taking something [that should there be because in one’s body one can never know, you cannot say I will look and tell that the viral load on this one is so much and maybe is transmittable to the other person. Rather you be on the protective side then you will say that continuation on that.

1:23 ¶ 96 in Clinic A - FGD

PARTICIPANT 287: Like PARTICIPANT 28 has alluded, discordant couples where maybe one of the partners is HIV positive and the other is not and they want to plan for kids, in cases where people that are not HIV positive, but want to… a person who is not HIV positive, but has a partner who is HIV positive.

1:26 ¶ 102 in Clinic A - FGD

PARTICIPANT 29: And to add on that, yes. Correct, and to add onto that, even if you may be dating a different partner they may be stipulate to you that they are had an exposure of previous sexual transmitted infection and of which if you had an exposure previously, you are most likely you can get that because it can be part of a lifestyle that somewhere somehow we pick up something somewhere as we go along and it just makes your system also be at a point that [they are very? HIV. It is something that can be another thing that can penetrate you because now we are ….

1:27 ¶ 104 in Clinic A - FGD

PARTICIPANT 29: Ja as they have already said it is those kinds of patients, I believe the kind of person who is in a committed, it may not be a marriage, possibility and we have witnessed that there is infidelity even in marriage. There can be infidelity in a committed relationship and it is not marriage at the time and even if you are both HIV negative, my advice would be that you can still take PrEP. PrEP may still be important because it does not matter how much you say you trust a person, things have happened. We have witnessed a lot of infidelity so for us to have that HIV free generation we can basically put each and every individual in the country on PrEP who is HIV negative.

5:1 ¶ 9 in Clinic D - FGD

PARTICIPANT 46: I also agree on that we do need PrEP because now if partners do come and, a couple come and do an HTS today and then we find out that it is a discordant couple, why not save the other partner and then the other one continues with the ART. So for me I think it is something that will work for...

5:3 ¶ 27 in Clinic D - FGD

PARTICIPANT 49: All right I believe it would be very good for like our nation because basically if I take PrEP knowing very well that it is going to work for me to actually prevent HIV then I am safe in my relationship, number 1.

6:4 ¶ 13 in Clinic E - FGD

PARTICIPANT 56: Yes, you know we recommend also even in adults, yes, both female and male, because mostly they will come and test, one will test positive and then one will test negative, so the negative one remains negative so we also recommend PrEP irrespective of age.

6:40 ¶ 113 in Clinic E - FGD

PARTICIPANT 54: PrEP supposed to be taken by people that actually have partners that are HIV positive,

---

○ Perceived benefits of using PrEP: 1.1 Young females control over sexual health. Prevention of HIV infections and deaths

19 Quotations:

1:8 ¶ 40 in Clinic A - FGD

PARTICIPANT 29: No what I wanted to say was that with PrEP, as much as I partly perceive it the way that PARTICIPANT 287… PARTICIPANT 28 perceives it, in the same breath I also believe that it is very vital to our generation. Looking at HIV, it is not something that you can end today, tomorrow or in the next 3 or 5 years, but if there can be a thorough understanding on what PrEP is, how PrEP works and the less the side effects we have on PrEP, we can achieve our goal for the next generation. PrEP can therefore eliminate HIV at some stage of life, whether we will be there or not but with PrEP I believe that HIV can be completely eliminated at a later stage. I am sure that there are illnesses that have been there that we have read about, but we no longer see so I think PrEP is one other way that will help to eliminate HIV.

1:9 ¶ 46 in Clinic A - FGD

PARTICIPANT 30: So my broader perception about PrEP is that we have made advancements in medication and I am concurring with PARTICIPANT 29 that we have come a long way with the prevention and treating of HIV. My perception is that like he said we have come to a point where we are preventing HIV and in the long run it will mean that we will have a generation that will not be exposed and also HIV positive

1:14 ¶ 56 in Clinic A - FGD

PARTICIPANT 287: It can be something that is viable for that person and can be user friendly kind of thing. A choice at the end of the day because you know you are looking at what can impact on me and something that, if we can go back to how much fear it brings to oneself finding out your status first and foremost and having to fall pregnant with the status and the discrimination that came with a lot of people that when you are labour ward with the status of being HIV, to most clients that I have witnessed in my past life in maternity it was, you know, that person felt lesser of a person than a person who is not.

1:37 ¶ 164 in Clinic A - FGD

PARTICIPANT 29: I think to somehow end this pandemic at some stage of our life. I think PrEP is basically leading us to that to have a generation that is free from HIV

1:55 ¶ 243 in Clinic A - FGD

PARTCIPANT 30: What encourages me to give PrEP is the fact that like we said we want to decrease the cases of new HIV cases and that would help in also preventing like we said other diseases that in HIV cases predisposes them to diseases, yes.

1:115 ¶ 166 in Clinic A - FGD

PARTICIPANT 30: Yes, PARTICIPANT 30, I agree with what he is saying. If we can end a pandemic that is basically exposing our people to many more illnesses and STIs, your TB because of being immunocompromised, so if we can deal with the one aspect definitely we will have a decrease in other cases or other diseases.

1:116 ¶ 168 in Clinic A - FGD

PARTICIPANT 29: We will improve the life expectancy generally of the country. The more we deal with … if you look at how many people die from TB who are HIV positive, if HIV is to be eliminated they will be having a better life.

1:117 ¶ 88 in Clinic A - FGD

PARTICIPANT 287: And you are planning babies as well. And maybe you are planning a baby and you want to have that thing that it is among us as Africans that if I have not given birth and maybe something that we cannot disclose to even our own families to say now PARTICIPANT 287 and PARTICIPANT 29, why are you not having babies like me? and him who are afraid of this HIV that is between us. You understand? Maybe the condom has mainly been the thing that we have been using but now where is the offspring from this marriage.

1:137 ¶ 20 in Clinic A - FGD

PARTICIPANT 29: She is somehow correct, although I am not entirely in agreement with what she is saying, in terms of saying that we are giving them a go-ahead to say go and have sex. Maybe yes, you are maybe giving a go-ahead of saying go and have unprotected sex, the reason being that no matter how…. Look we have talked about condoms for as long as we can remember the HIV pandemic, but still the condom is not used.
PARTICIPANT 29: So now that we know that there's PrEP and PrEP prevents us from this deadly virus that we are scared of which is HIV, why should we still use condoms. So we are going out there, we have PrEP, we are sleeping around because we know that we will never ever catch HIV,

3:1 ¶ 5 in Clinic B - FGD

PARTICIPANT 31: I think PrEP is needed in our communities and clinics because it's a prevention that it's going to help young people, young girls especially to prevent themselves from getting HIV.

3:2 ¶ 11 in Clinic B - FGD

PARTICIPANT 37 Ja, I think it's going to help again to the reduction of HIV infections.

3:3 ¶ 18 in Clinic B - FGD

PARTICIPANT 36 Ja, my feeling is because remember you said that we wanted HIV free generation. The only way to get there is through medication because we've tried the condoms, they're still not working.

4:1 ¶ 11 in Clinic C - FGD

PARTICIPANT 39Maybe I am a little bit biased because I have worked with, from 2004 I was working with kids. Kids with HIV, on ARV's, teenagers and so on. I would rather not see young women on ARV's. There is something that they can do to protect themselves and protect their health. Yes, we have ARV's, but we still have opportunistic infections and stuff. But if they can protect themselves and know that they are on PREP, and know they are protected.

4:2 ¶ 13 in Clinic C - FGD

PARTICIPANT 39Yes, we have ART's, but you know, HIV comes with a lot of things. Others becomes sick, we have opportunistic infections. It is not only about them taking ARV's and that. We know that there is a virus that can cause a lot of problems and issues. So, if they are on PREP, they are HIV negative, they are protected with PREP, they use condoms. For me, I think it is important.

4:37 ¶ 84 in Clinic C - FGD

PARTICIPANT 40: Education, education, education. Because now, it is better, like participant one has said, it is better to prevent than to cure. Yes, so, we are just like family planning. We will always have these, but maybe I think we will a certain, a lower percentage of those who believe … even I, I cannot take that, just like family planning. There will be, it makes me fat and what, but it is there to prevent teenage pregnancies. Now PREP comes today, it is there to prevent HIV positivity. So, why do we have to stigmatise it, because there is nothing wrong.

4:60 ¶ 200 in Clinic C - FGD

PARTICIPANT 42 I would say that PREP is a treatment to prevent HIV.

5:4 ¶ 33 in Clinic D - FGD

PARTICIPANT 50: I am going to agree with Participant 4. I am agreeing in a sense of PrEP prevents you in all odds for one to being infected. Yes. Sometimes we do things without thinking about the consequences. Especially nowadays. So for my side I say like all youth should use PrEP as long as they HIV negative.

6:1 ¶ 3 – 5 in Clinic E - FGD

PARTICIPANT 53: Okay, we need PrEP because it is helping us, especially for our younger females, yes, they need to have protection.

INTERVIEWER: Uh-uh, so you are saying we need PrEP for young females to protect themselves; what do you mean when you say to protect themselves, protect themselves against what?

PARTICIPANT 53: Against HIV, yes.

6:2 ¶ 7 in Clinic E - FGD

PARTICIPANT 54: I would say it is very true, we actually do need PrEP because a lot of people honestly, people [do not really use condoms and they do not really know the other person's status, so PrEP will help prevent the increase of people that are actually having H- ... that are HIV positive.

○ Perceived facilitators to PrEP uptake among females: 3.1 Healthcare provider facilitators. Healthcare providers' age and competency

16 Quotations:

1:15 ¶ 62 in Clinic A - FGD

PARTICIPANT 28: Yes I am trained on PrEP but I do not have a certificate.

1:16 ¶ 64 in Clinic A - FGD

PARTICIPANT 28: PrEP is an antiretroviral that contains 2 drugs that you need to take at least 7 days before exposure to HIV.

1:17 ¶ 68 in Clinic A - FGD

PARTICIPANT 29: Ja, I have been in sales on what is PrEP and how to give PrEP as she has explained. It is an antiretroviral that has if I am not mistaken, so it is a pill that you take daily for the longest of time, for as long as you think or you feel that you may be exposed to HIV, not necessarily only on sexual intercourse, it can go as far as drug use, as far as being involved in car accidents?, it just highly prevents HIV infection so ja, that is briefly what I can give.

1:18 ¶ 72 in Clinic A - FGD

PARTICIPANT 287: No, as far as I can say to know the? information, I can go to as far as the information that was given to me and back, I am trained

1:19 ¶ 74 in Clinic A - FGD

PARTICIPANT 30: PARTICIPANT 30, I am not trained, PrEP trained, but I do have just a little information on what PrEP is

1:20 ¶ 84 in Clinic A - FGD

PARTICIPANT 30: It would have been enough that kind of concentration in your body and moreover, so to add to what PARTICIPANT 28 was saying, the 7 days part of it is to also go as far as emphasising that it does not only end there. The medication in itself, the lifespan in the body is a 24-hour cycle, it needs to be renewed every 24-hour cycle in order the peak of protection, it does not subside and then should there be any loophole it cannot find that gap, it is like making but building blocks it needs to just be continued.

1:134 ¶ 444 in Clinic A - FGD

PARTICIPANT 28: They are more open to me. They are actually more open even when discussing other sexual things. They are actually open to me, they will actually be laughing. They will be like Sister imagine if it was older nurses I would not be saying these things so I really fully agree with her.

1:135 ¶ 446 in Clinic A - FGD

PARTICIPANT 29: Ja I am also in agreement with what PARTICIPANT 30 has said. I am dealing with males and it is here they do say to me on so many occasions that hey imagine if it was a woman. So that touches on this that at times a person looks at who am I talking to, can I be free with this person, is this person not going to judge me, is she not going to act like a mother to me, can we be friends and we talk like friends with this person so that can improve it.

3:41 ¶ 359 in Clinic B - FGD

PARTICIPANT 36 I have been trained.

3:42 ¶ 363 in Clinic B - FGD

PARTICIPANT 33 Yes, I have knowledge of PrEP but I have not practised it.

3:45 ¶ 373 in Clinic B - FGD

PARTICIPANT 35 I have been trained.

3:46 ¶ 375 in Clinic B - FGD

PARTICIPANT 31: I have been trained.

3:47 ¶ 379 in Clinic B - FGD

PARTICIPANT 37 I know everything about it, but I was not trained.

5:31 ¶ 117 – 127 in Clinic D - FGD

PARTICIPANT 46: Yes I am.

INTERVIEWER: Participant 2 are you trained?

PARTICIPANT 47: Yes.

INTERVIEWER: Are you trained Participant 3? Participant is not trained. Participant 4 are you trained?

PARTICIPANT 49: Still training.

INTERVIEWER: You are still training or you have trained, or you are being mentored?

PARTICIPANT 49: Trained.

INTERVIEWER: You are trained. Participant 5?

PARTICIPANT 50: Mentored.

INTERVIEWER: You are mentored. You are not trained, but you are mentored. Participant 6?

PARTICIPANT 51: I am trained.

5:50 ¶ 129 in Clinic D - FGD

PARTICIPANT 51: Okay when it comes to the attitude that is why they place youth ambassador because they relate that is why they also placed a young nurse because they are friendly to them.

6:48 ¶ 142 in Clinic E - FGD

PARTICIPANT 54: With me because I am working with the sisters that actually know about PrEP, so if I am having any difficulty it is just about walking out of my room to the next one and just asking about it.

○ Perceived facilitators to PrEP uptake among females: 3.1 Healthcare provider facilitators. Indications for PrEP initiation

6 Quotations:

1:58 ¶ 251 in Clinic A - FGD

PARTICIPANT 28: encouraging or discouraging, if a patient comes who wants PrEP, is making an informed decision, is meeting the criteria of 15 to 24, I give.

1:59 ¶ 255 in Clinic A - FGD

PARTICIPANT 28: No, as long as I give you the correct information, the patient is aware of what I am saying and understands and she wants it, I am going to give.

1:60 ¶ 259 in Clinic A - FGD

PARTICIPANT 29: 3, encouraging me would be that now I know this 15-year-old meets the criteria so there is no reason for me to not give

4:86 ¶ 266 – 272 in Clinic C - FGD

PARTICIPANT 44 If we test you and we find you HIV negative.

INTERVIEWER: So, if we test you, as long as we test, we want you to be HIV negative.

PARTICIPANT 44 And you tell us that you are sexually active. Because you can put if you are a person who-

INTERVIEWER: Yes, so you test, you test negative, and you are saying when they are sexually active, they are eligible for PREP, meaning that person you will offer the medication.

PARTICIPANT 44 Because we test for pregnancy before we start.

INTERVIEWER: Not pregnant.

PARTICIPANT 44 Test for pregnancy, we test for HIV.

5:37 ¶ 154 in Clinic D - FGD

PARTICIPANT 46: The client who is eligible for PrEP is one who has tested HIV negative and then also pregnant women they are eligible and also it includes the breastfeeding ones, they are also. So males and females, all ages.

5:38 ¶ 156 in Clinic D - FGD

PARTICIPANT 48: I would say everybody, who has tested HIV negative and is ready and willing to take PrEP after being explained about it?.

○ Perceived facilitators to PrEP uptake among females: 3.1 Healthcare provider facilitators. Interventions to destigmatise PrEP use

5 Quotations:

1:36 ¶ 152 – 158 in Clinic A - FGD

PARTICIPANT 28: What I have done before I have asked my patient to talk to her partner and to disclose and if the partner still does not understand, she can come with the partner so we can have an open discussion together that he can understand

INTERVIEWER: Oh so you are inviting the partner?

PARTICIPANT 28: Yes.

INTERVIEWER: So that they can come together with the patient so that you can explain, just to reduce that stigma.

PARTICIPANT 28: Stigma, yes.

INTERVIEWER: Oh okay and is that working for you?

PARTICIPANT 28: It has worked. That was the only patient that we managed to retain

3:53 ¶ 418 in Clinic B - FGD

PARTICIPANT 32: Like if they recruit, they recruit young ones for PrEP and they give information about PrEP and direct them to the relevant places. district they are called PrEP ambassadors.

4:22 ¶ 59 in Clinic C - FGD

PARTICIPANT 44 Yes, I did have maybe four or five patients, who said, you know, my sister, or my cousin … as you know, we stay in extended families. My aunt, my uncle is taking the ARV's and the bottles are blue, like our bottle is blue. So, I used to tell them that it is not a problem. Simple, you take another bottle, maybe a white one, decant the….

4:23 ¶ 65 – 67 in Clinic C - FGD

PARTICIPANT 44 Because you stay with Gogo, malume You take an empty container, a small bottle.

INTERVIEWER: So, you would advise them?

PARTICIPANT 44 Yes, because some of them have the challenge which I explained. They do not want other family members to know that they are sexually active. They are hiding that . So, I will simply say, there are many small bottles everywhere. You decant, put it in your comfortable place, take it and no one will ever know that you are taking it.

4:24 ¶ 69 in Clinic C - FGD

PARTICIPANT 42 No, I agree with her. That is a good strategy. Because now, even at the pharmacy, they are giving us empty packets, so we can just put it in there, and not really write a name, just write the name of the patient, but not write the name of the medication.

Although you will still write the batch number, the expiry date, but not write the name of the medication, then only you and the patient will know what-

---

○ Strategies to improve PrEP uptake and retention: 4.1 Strategies for health care system. Nurses competent to provide PrEP services to youth

10 Quotations:

3:14 ¶ 115 in Clinic B - FGD

PARTICIPANT 31: Maybe lack of information, if I was told, given enough information about this PrEP. It would be able for me to encourage the professional nurses to go and train for that, so that we are able to treat each and every little, the girls who comes in.

3:59 ¶ 432 in Clinic B - FGD

PARTICIPANT 32: Lets say its not, it could be off on every consultation. Everyone could be trained about PrEP, get the correct information about PrEP and redirect the patient to the right….

4:12 ¶ 124 in Clinic C - FGD

PARTICIPANT 40: What I am saying is we can do peer teaching. Not all of us can go for formal training. There are many departments here, like admins, they cohort. We are just doing peer, one orientating another.

4:40 ¶ 122 in Clinic C - FGD

PARTICIPANT 39If it end up with people caring programmes, it is like, we know it sister, it is sister so-and-so. When that person is that on duty, or on leave, it is a crisis, because we have … training does not mean you have to go in a formal setting. We can do in-service training, informal in-service training, where we update each other, regularly

4:58 ¶ 366 in Clinic C - FGD

PARTICIPANT 39Training of health professionals. It does not have to be in a formal setting. Informal training, ongoing, yes.

6:11 ¶ 23 in Clinic E - FGD

PARTICIPANT 54: and also, the other thing is I also think that we as clinicians, we also need more training when it comes to PrEP and everything

6:17 ¶ 39 in Clinic E - FGD

PARTICIPANT 55: I think continue to train them. So, we are expected to they are expecting you to start giving PrEP. So if in two days train for two days. Yes, so if…

6:23 ¶ 72 in Clinic E - FGD

PARTICIPANT 56: Okay I agree with her, but we need to be trained, all of us, so like if I come across the patient who needs PrEP, I can be able to prescribe or to teach or to help the patient without sending her to specific Sister, even that I think it is going to help a lot, because here in our clinic, I think we are only three or four, yes with us, we are only four of us who were trained in PrEP. So if we can train all of us, all clinicians it can also help.

6:39 ¶ 148 in Clinic E - FGD

PARTICIPANT 53: If the client or patient came with like a little knowledge, it is easy to give or to initiate, if the counsellors they can be trained, more trained, because here yes, who are working with the counsellors.

6:46 ¶ 134 in Clinic E - FGD

PARTICIPANT 54: I think the only thing that will come from them, we just need more staff, more staff, yes, all that and then training on top of that.

○ Strategies to improve PrEP uptake and retention: 4.1 Strategies for health care system. Nurses dedicated to provide PrEP services to youth

13 Quotations:

1:98 ¶ 374 in Clinic A - FGD

PARTICIPANT 30: On the health systems side is like we have alluded to the long queues, if maybe if there was a section that was specifically concentrated to PrEP patients where it is convenient for the patient to come at a particular time where the professional has planned or scheduled to see this number of clients where it would reduce the time and they are seeing the same person who they have built a rapport with or also if it would reduce them coming physically to the clinic. I do not know if we have got systems like your short left for patients who are on ARVs and other chronic medications. If maybe we had a system that would work with… there are people that are going to school that we focus on and there are people that are working, maybe it would work around their schedules, also

1:108 ¶ 440 in Clinic A - FGD

PARTICIPANT 30: Another thing is because we are targeting an age group from 15 to 24, they are more likely to comply if it is a person or the clinician that is seeing them is around that age and does not… the perception is that the person does not look like a father or a mother. I think also if it is a relatable thing that will have a bigger uptake.

3:55 ¶ 424 in Clinic B - FGD

PARTICIPANT 36 I think the fact that we are dealing with young people right, so you need to have a friendly, user friendly services, services whereby they are not judgmental. Because we have talked about being judged that you are having sex and all of that. So having young people working in that facility that you service will make me feel comfortable to come and talk to me about PrEP so I feel more motivated because I know there is a person that understands me there and who will give me, of course will give the information but it feels on how you're giving me the information but if I tell you, if you take this and I make you understand why you are taking it, it becomes more easy.

3:63 ¶ 442 in Clinic B - FGD

PARTICIPANT 36 That is correct, in a certain district I worked with, the PrEP was doing well you can check yourself. In every facility needs to have PrEP in the morning they come in, their banners are up from the gate. Then their tents are ready, and every young person that comes in is screened to say come let me tell you about PrEP and they talk to them. There's enough stuff to do, that's already when they come in the gates they are already being given the information. Then they come in and they are redirected to where PrEP is. When you get there is colourful, its TV's, its WIFI's, its friendly. The nurses there are ready, they are young, they have their shirts on, they have got their caps on you know, when you get … and the sisters we are ready and I'm ready for when the kids come in. They are there, someone will come during the day and they knock off and the other one comes in. They are given those services and PrEP used to run well, so that's where I saw it working well. We can all be trained but when you start at the gate and redirect to where they are supposed to, they even had footsteps where PrEP is, sop just follow the footsteps to see where youth friendly service is. So I think that was a good initiative.

3:64 ¶ 444 in Clinic B - FGD

PARTICIPANT 36 Give them t-shirts, there is nothing more nicer than being given a shirt, skipper, you give them T-shirts, call it a skipper. You give them T-shirts with different colours, they come in, you get this shirt it goes with a cap, follow, then there is someone taking you there to go and see. The sister is very happy, you are welcome, you know this is what we do, there is Wi-Fi, there is TV, there is everything, there's pamphlets, you know there is everything.

3:65 ¶ 455 in Clinic B - FGD

PARTICIPANT 36 Jeans, tekkies, not being formal with your read epaulettes.

5:34 ¶ 150 in Clinic D - FGD

PARTICIPANT 51: So the solution to this is that there should be PrEP clinic, a clinic for youth, like there was supposed to be an ideal clinic for the youth to come and do their PrEP and the other services. So that is the only solution whereby the will meet youth friendly staff so that they do not have to, so I think it will be better if or if it is actually not the same clinic as the? a section that is youth friendly showing we are providing youth services, PrEP; so that they can come back. They do not want to come this side and come and be with the other, that also ticks them off, it makes them like [rather go away?.

5:36 ¶ 243 in Clinic D - FGD

PARTICIPANT 46: And then also I am seconding participant 4 by saying that they should have their own corner whereby we will know, they go there for most of the services, not only PrEP. They cover whatever they come to the clinic for and if everything is under one umbrella I think it will work for them.

5:54 ¶ 254 – 257 in Clinic D - FGD

INTERVIEWER: So really this thing of having elderly people, you are discouraging it?

PARTICIPANT 46: Not at all They must not be there at all, because okay we are going to make a user friendly, youth friendly service, that has everything, has family planning, but when the child comes, a 14-year old

INTERVIEWER: So the elderly, [the problem to work with youth, they must be out?.

PARTICIPANT 46: Like myself , please do not train me…

6:24 ¶ 74 in Clinic E - FGD

PARTICIPANT 54: It is very true, often patients from pillar to post, but then again if you have ... because what I know is we are supposed to holistically, you understand, so if I am having a patient, I am supposed to treat that patient for everything. If I am then I should be able to do everything and go with the patient there so that if that person, the next person, I am still [dealing with this, then I can also know where to. Instead of just sending the patient alone because then they will be questioning her, oh but why did you not get help here and then starting to not get what the patient is there for or whereas if you just accompany the patient, then it will be also better, yes.

6:25 ¶ 76 in Clinic E - FGD

PARTICIPANT 54: It will also depend, if ever the whole line, all the people are for PrEP, then it is going to be a bit of a problem, at that point they should be having like Participant 3 was saying, that at least have a place where there is only one nurse who is doing PrEP, but also with that one person who is doing PrEP, we also have to have other ones, that when this one is absent, not on duty, then somebody else can help. So I am not really expecting to the point uguthi when I am seeing a patient, then all of them are going to be saying they want PrEP and I have to take them, so I am not really expecting them to always like, in the queue there is going to be PrEP, PrEP, PrEP, because I would like to think uguthi? somewhere, somehow, when they came into the clinic, somebody has to

know this person came for what. For whatever they came for, that the person already ... the person's in nursing also told them about PrEP and then this person is saying yes I do want PrEP, then they should send them to that person who gives them PrEP and then treat them holistically.

6:45 ¶ 131 in Clinic E - FGD

PARTICIPANT 6: What will usually happen is, if you are not understanding PrEP and all of that, that is why we just this person needs PrEP, this person is doing PrEP at the moment, so we will just say to that person, not asking questions or anything, just like oh, so you are here for PrEP, you also need information on PrEP, please go talk to this particular person. At the end of the day you are sending only the patient and you do not know, you still do not understand or not you also will not know what happened to the patient did they actually get initiated or they were like no because of pregnancy or because of certain reasons, no we did not.

6:49 ¶ 66 in Clinic E - FGD

PARTICIPANT 55: Hire more let say for example with PrEP, we know so this is dealing with PrEP, even so now doing everything from and all those things, so we end up so if we have more staff.

○ Strategies to improve PrEP uptake and retention: 4.1 Strategies for health care system. Nurses with a conducive and helpful attitude

8 Quotations:

1:12 ¶ 48 in Clinic A - FGD

PARTICIPANT 28: And of which PrEP at the moment because it does not have anything that holds you, even if you can give information to the client to say you can take a break on that moment, it is not that you can take a break from family planning. So does PrEP, you can take a break from that moment and also when now you see that, I have seen PARTICIPANT 29 now I want to go back now and do that thing again. Then I can go back to PrEP and I am welcome back despite the fact of being given that very same so that you can try by any means to retain them and telling them that they are also allowed to have that breather, that break against what you are saying about being fatigued against treatment and come back now when you are now back onto the [infidelity activity, you understand?

1:75 ¶ 289 in Clinic A - FGD

PARTICIPANT 29: It is more or less the same as what she is saying about I know that this coming weekend it is going to get rough and I heard there is a pill that prevents HIV, that I got and I am looking for that pill that will help me be safe in this roughness that is coming

this coming weekend. So it takes me to that, if there was that kind of pill or that kind of whatever that you could take as a prophylaxis just before and knowing that after I have taken this pill I can be a rough as I want for the next 3 days and I do not have to take this pill each and every day, we will … I am sure there will be a difference.

3:15 ¶ 141 in Clinic B - FGD

PARTICIPANT 37 Okay we usually try to reduce the visits at the clinic. Like when they come and I needs to take bloods. Then it's going to be one month follow-up. But after that we combined it with medi planning and then they come every three months to the clinic. So maybe they'll be coming four times in a year

3:16 ¶ 143 in Clinic B - FGD

PARTICIPANT 37 I don't think it's in the guidelines but we're trying to assist patients.

4:48 ¶ 422 in Clinic C - FGD

PARTICIPANT 43 In as much as we are promoting health, by taking those bloods and checking the results, but I think if we could come up with a strategy of reducing the number of times that we test a patient. I mean, it is too much. Even when they are positive, they are tested once a year. Here am I, I am fine, I am supposed to be tested every three months, it is too much.

4:56 ¶ 378 – 380 in Clinic C - FGD

PARTICIPANT 39Yes, she is going back to what I said earlier, we must build good interpersonal relationships, we should be at their level, be able to talk, be able to-

INTERVIEWER: To talk their language.

PARTICIPANT 39Yes, be at their level, so just be non-judgemental.

4:57 ¶ 376 in Clinic C - FGD

PARTICIPANT 40: It is not really new, it is just that we have to be non-judgemental. Because when a young person comes and says PREP, and then you start by saying, at your age. And then that is the attitude. We must try to be non-judgemental.

5:46 ¶ 211 in Clinic D - FGD

PARTICIPANT 47: I am saying we are nurses, but again we are also mothers, so now that we know that PrEP can protect these children, then you know as a mother you will do anything to protect a child. That is what motivates you.

○ Strategies to improve PrEP uptake and retention: 4.1 Strategies for health care system. PrEP accessibility

11 Quotations:

1:67 ¶ 481 in Clinic A - FGD

PARTICIPANT 29: And make it available in all facilities.

1:69 ¶ 465 in Clinic A - FGD

PARTICIPANT 287: That is why that one is being done right now, but the thing is the access the client that regardless of where the client was initiated the client can go anywhere where else where it is nearest and collect.

1:70 ¶ 462 – 464 in Clinic A - FGD

PARTICIPANT 287: It may be accessible to every clinic but the main thing is that with where the client was initiated, the client has defaulted on my part. She may be continuing on the other point and everywhere she is a new case.

INTERVIEWER: So you are saying there has to be a link between clinics …

PARTICIPANT 28: Or something like…

1:71 ¶ 460 in Clinic A - FGD

PARTICIPANT 287: Okay there is and then maybe you will find that maybe the client is having a challenge to get the PrEP. So basically this PrEP it needs to be in every area that is reachable to the client and it does not limit a client to be only at this facility. I can go, let us say I initiated here and then next time I have gone to whatever place and it is far away from where I initiated, I can go to her and I can collect the PrEP. It can also keep the client within you know…

1:99 ¶ 394 – 398 in Clinic A - FGD

PARTICIPANT 287: If let us say [not the client outside they have gone through the processes of being initiated and so forth, cannot it maybe be done like other chronic medications?

INTERVIEWER: So …

PARTICIPANT 287: Be collected at whatever

INTERVIEWER: External pickup …So basically what she is saying, she is proposing the canteen.

PARTICIPANT 287: Ja.

1:111 ¶ 450 – 454 in Clinic A - FGD

PARTICIPANT 29: No I am aware of that. I am just… look it is for the purpose of the study I am focusing on the age that we are focusing on, I am not limiting this to any other age group, but I just want to come with an awareness that as much as we are focused on this age in this particular study we should also remember that this age group you will find a 15-year-old sleeping with a 47-year-old and when we go out there trying to say you know what guys we have a challenge with this age group. I know because of the new positives that we find, they are mostly within this age group, but where do they get it from? Do they get it from the very same age group?

PARTICIPANT 28: Their own age group, no most likely-

PARTICIPANT 29: They get it from the older people? So as we approach the PrEP I wish it could be general instead of focused on a certain age group.

INTERVIEWER: So that it includes the next person that you are actually exposed to their HIV so you are saying let us include everyone when it comes to PrEP.

PARTICIPANT 29: The same 15 to 25-year-olds are giving HIV to the older ones. So the older ones also need protection from the same 15 to 24 so as we go out, let us be broad. Let's be general about

1:113 ¶ 458 in Clinic A - FGD

PARTICIPANT 287: So how am I going to give you that support that you may need, let us say like telephonically if you gave me a wrong number. Who am I to reach out to? So it also boils down to the very same thing that you know in as much as we have been giving information to clients I do not know whether should I say or would I be correct if whatever sentence that is given to the client based on that one particularly, it should not discriminate where I am or where I stay because most of the other areas they do not necessarily have PrEP. I am coming from Protea something it does not offer PrEP. I come to Dobsonville, I have got clients that come from that far asking nana, this far, but this is where I can get PrEP.

3:35 ¶ 337 in Clinic B - FGD

PARTICIPANT 34 As much as we're saying, as much as we're expecting stats from this five-clinic plan A, B, C, D and E you should also I think ... One step from the very facilities that are not coming up on board.

3:36 ¶ 341 in Clinic B - FGD

PARTICIPANT 36 Lets evaluate everyone who is now given a way go ahead to continue implementing PrEP. So let's have the demand from these people.

3:57 ¶ 428 in Clinic B - FGD

PARTICIPANT 31: Maybe if PrEP could be offered in all health department maybe that could help, if they could take PrEP.

5:43 ¶ 221 in Clinic D - FGD

PARTICIPANT 50: Let them actually make sure that they actually create a user friendly? in each and every clinic across South Africa and make sure that they are actually resourced with all these particular resources that we actually need to run this particular programme. Then I believe we can actually achieve the HIV free generation that we actually talking about.

○ Strategies to improve PrEP uptake and retention: 4.2 Strategies for information dissemination. Information dissemination at different locations

9 Quotations:

1:106 ¶ 435 in Clinic A - FGD

PARTICIPANT 28: If possible even go into the high schools and tertiarie

4:7 ¶ 468 in Clinic C - FGD

PARTICIPANT 44 Yes, we should even have it at the malls, where they like, another place they like, the malls. The stadiums, the grounds

4:8 ¶ 464 in Clinic C - FGD

PARTICIPANT 42 Human resource, we need participation with the school health, the promotors and the, yes, all those stakeholders that we can get a hold of.

4:82 ¶ 248 in Clinic C - FGD

PARTICIPANT 39Maybe in school health, the school health nurses, they should be involved.

4:98 ¶ 346 in Clinic C - FGD

PARTICIPANT 43 We also need programmes like outreach, outreach programmes where, besides school, they go into the communities to educate, to campaign, to promote all these things.

4:99 ¶ 350 in Clinic C - FGD

PARTICIPANT 44 Yes, health education, we should also involve the churches. Also, we should involve the taverns, shebeen queens, because at the end of the day, there places where you will find these kids drinking, like, I do not know. So, you must involve the owners of the shebeen queens, taverns, those places.

4:100 ¶ 352 in Clinic C - FGD

PARTICIPANT 44 And it would be very effective, especially with taverns. Because there they are so relaxed. When you are relaxed, you listen. Anything they say, whether it is place, there are nice stuff that they like, definitely they will listen.

4:102 ¶ 360 in Clinic C - FGD

PARTICIPANT 45 So, they are leading our communities. And also, like those people that are closer to our communities and the traditional healers, chiefs and the like, they have that belief of, what is this facility testing and stuff like that. Then to say, they are still virgins and stuff like that. So, we need to involve all those people, just to ease their minds, to say, anything can happen, even if the young lady is a virgin and stuff like that. So, they must know about this thing and give education to them.

4:103 ¶ 364 in Clinic C - FGD

PARTICIPANT 42 No, I support what he is saying about the traditional healers because even the counsellors, they can be involved, and that would make a big impact. Because they are the face, and their voice also. These young children will definitely listen to them, because they are our leaders.

○ Strategies to improve PrEP uptake and retention: 4.2 Strategies for information dissemination. Information dissemination in a conducive way

9 Quotations:

1:13 ¶ 48 in Clinic A - FGD

PARTCIPANT 28: So it just boils down to the level of understanding, the information that needs to be out there for everyone, not only when something now biting onto the flesh of people and it is like it is being imposed on people. It needs to come as information first so that when you are making that option you are making it with a broader mind knowing that in as much as saying I am preventing myself from falling pregnant and I am not ready, I am still a child so does PrEP, it needs to come as that kind of information, not to say that there’s anyone who is compelled to be on it at that moment because it is not everyone who is going to take the very same family planning.

1:47 ¶ 193 in Clinic A - FGD

PARTICIPANT 29: Yes, that can be an argument of then depending on a person that you know what I am still searching. You educate the person, you give her the options and say look if you want to take PrEP, then we give PrEP for this purpose, for these reasons. If you want it whether you are active at the time or not, we will give you.

1:107 ¶ 438 in Clinic A - FGD

PARTICIPANT 287: Because to some of the clients that even if you can say what do you know because basically when you interview a client before you can indicate or anything you move from the known to the unknown. You first find out from the client okay fine you came for PrEP, what is it that you know of PrEP, what did you hear about PrEP? So that at least to certain information that you can go to whatever extent in explaining to this client is not going to be repetitive for this one, it is going to boring this person. You move from the known to the unknown and clearing out the grounds. By the time that person leaves the person is sorted

3:56 ¶ 426 in Clinic B - FGD

PARTICIPANT 38 Free information. Give them before they go to the sister, into the consultation room, once they have got the information outside, when she comes into the consultation room the sister reinforces the information that they got outside and then that will make them understand.

4:9 ¶ 450 in Clinic C - FGD

PARTICIPANT 42 Yes, but we still continue just by encouraging them, even though there are no refreshments, we can just encourage by word of mouth.

4:15 ¶ 31 in Clinic C - FGD

PARTICIPANT 44 With me, I support PREP 100%. I did work there, although I did not go to the workshop or school, to be taught about PREP, but I was orientated by the sister from Hanover. So, I practise it, and I was allocated there. To me, PREP was doing well at first. I support it 100%. These kids, we need to create a good relationship with them, because I was dealing with youth. Dealing with youth, some of them are sceptical, some of them are junkies, some of them do not trust us, some think that you might talk about them. So, if you build a good relationship and they are used to you, you make them your friend. You befriend them, you go to their level, you talk their language. Then they tend to their, they will come. Like I was working with a sister from Hanover, we did that, made them our friends. And we used to make them, say anything they want to say, as if we were all the same age group. And they used to open up.

4:19 ¶ 39 in Clinic C - FGD

Yes, an interpersonal relationship with them, be at their level, talk their language.

4:20 ¶ 43 in Clinic C - FGD

PARTICIPANT 42 Yes, coming down to their level, as sister is saying, talking their language, that is the first thing. If we can do that, we will win this. And not being judgmental as well, yes.

5:48 ¶ 172 in Clinic D - FGD

PARTICIPANT 47: We actually, it is like initiating , you cannot think that the educator said everything. You also do your own education. Even if you know they told the person daily, but what if they forgot. So you actually start afresh, to say daily you must eat first, you must use condom, we actually do it.

○ Strategies to improve PrEP uptake and retention: 4.2 Strategies for information dissemination. Information dissemination through different media

24 Quotations:

1:68 ¶ 477 in Clinic A - FGD

PARTICIPANT 287: That is true, because even now you can actually even switch on your radio, the reminders that are going on, on a daily basis. I do not know maybe you did hear this when you were driving this side, you heard they are reminding ‘go vaccinate, guys’. Go what-what guys. That one, in as much as they want out as much to heal this thing that is killing people and this one too, they might as well heal because I believe those who are out there who gets to find maybe information pertaining on how to care for whatever ailment that is how fast it can be dealt with, let them do it the very same way they dealt with Covid.

1:101 ¶ 404 in Clinic A - FGD

PARTICIPANT 30: I feel that the Department in terms of media coverage and education on our smartphones we got, people google and we are targeting kids that are 15 to 24, who are on social media but the Department is not as active on media [of the very? people that are being targeted are concentrated on. So in my opinion information should be targeted to where the group that they are targeting is focused on.

1:102 ¶ 406 – 409 in Clinic A - FGD

PARTICIPANT 28: TikTok, Facebook.

PARTICIPANT 29: Twitter.

PARTICIPANT 30: Twitter is too much English there. You must make it interesting for me to want to look

PARTICIPANT 28: The kids are there. The 15-year-olds are on the Twitter. you must remember information if you were bringing it to me through a video, an exciting video I would want… it would catch my attention and if you want me to read something, imagine having to read that thing. I am going to get bored.

1:103 ¶ 411 – 416 in Clinic A - FGD

PARTICIPANT 29: No, but we are not limited it, we are saying even to Twitter, there are people who just love Twitter you will find me, I am hardly on TikTok. I do not know when do I go to TikTok. I get those videos through WhatsApp, through this and this, but a day will not end without me taking Twitter and I find long statements on Twitter. I sometimes read them.

PARTICIPANT 28: Yes, you read that but remember that age group from 15 to 24 do not have time to read. I have got exams to read

INTERVIEWER: Ja, so but …

PARTICIPANT 28: to send me a text message on WhatsApp that is too long, after 2 sentences, bye-bye.

PARTICIPANT 29: No we are not saying it should be the only ways?

PARTICIPANT 29: TikTok if we realise that TikTok is used mostly by this age group, there may be 1 or 2 who are using Twitter and who do not like TikTok.

1:104 ¶ 424 in Clinic A - FGD

PARTICIPANT 29: If men’s clinic? can be advertised on TV, why cannot they put PrEP there and say guys there is PrEP.

1:105 ¶ 430 in Clinic A - FGD

PARTICIPANT 287: In as much as there are still those despite the fact that there is all billboards of HIV and what-what, how does one contract HIV? There are still those that are actually, they blind themselves against the knowledge but at least an attempt into reaching out through the very same platforms that can be user friendly for all of those that know themselves to be you know.

3:27 ¶ 223 in Clinic B - FGD

PARTICIPANT 36 Advertise, social media, going to schools, school campaigns, I think that would.

3:62 ¶ 438 in Clinic B - FGD

PARTICIPANT 37 I think another thing if we can make out this is more attractive. Try to make research on what is it that the young one's like] what is what they once like. It's like maybe if the Department can have a Wi-Fi then they will attract more. Many will say there is Wi-Fi there and so many will be coming.

4:78 ¶ 230 in Clinic C - FGD

PARTICIPANT 40: We are dealing with youth here who are always on social media. We can use social media, most, because, yes.

4:79 ¶ 232 in Clinic C - FGD

PARTICIPANT 42 And also on TV, advertised on TV, just like the ARV's. You see, when the ARV's come, say the child is on that treatment, the child will say, mommy, that is my treatment, they can point it out. So, we need to do the same with PREP, so that the community will be aware, and there will no more be a stigma.

4:80 ¶ 234 in Clinic C - FGD

PARTICIPANT 44 Just like that advert whereby the Gogo and the grandchild … Gogo, apparently is like, she saw the grandchild going to , and then Gogo congratulated the grandchild, saying that no, do not be embarrassed, use dual protection. That advert, I wonder if you know it? Use dual, it is like condom and method. Condom and method. If they can do the same, so that they should be on the know that HIV can be prevented, or rather to take the PREP.

4:81 ¶ 236 in Clinic C - FGD

PARTICIPANT 44 Media is the best platform.

4:97 ¶ 338 – 340 in Clinic C - FGD

PARTICIPANT 40: Not to only the youth, because the youth, as far as I understand, they are still dependant. So, education, education to youth and communities for their understanding. And then-

INTERVIEWER: So, before you go to number two, so, how do we give health education, this education to the communities?

PARTICIPANT 40: Like we said, social media, TV, whatever, the billboards, whatever we can come up with.

4:101 ¶ 356 in Clinic C - FGD

PARTICIPANT 42 Leaflets that we can give out here, if the patients are sitting, we can just hand out the leaflets and say, there is something called PREP.

5:16 ¶ 65 – 67 in Clinic D - FGD

PARTICIPANT 50: And the other thing I think that we should try and communicate them through cell phones.

INTERVIEWER: Uhhmmm so is it going to work on the cell phone?

PARTICIPANT 50: Maybe call them or use their Whatsapp contact.

5:17 ¶ 73 in Clinic D - FGD

PARTICIPANT 46: I think that can also work if there is a communication between the parent and the client that has been initiated. And the other thing, though I think that it has been done before whereby we go to the media where it will promote, because now I think if it goes to a media to say that here is something that we have it is being done maybe for only that month and it is not something that is continuous because now we cannot only rely on parents who come for chronics and acute here, because there are some who do not even come to the clinic, they do not attend the clinic. So how do we reach them? By reaching them is via media.

5:18 ¶ 79 in Clinic D - FGD

PARTICIPANT 47: I think we can use study material like hard copies of all the information about PrEP. Then they read at the comfort of their own home in their spare time, because sometimes they are here they are sick, they will not even listen to me, because they came for their own problem, but if you give you a pamphlet in the comfort of their own home in their own spare time then they read, they will understand. Most of them are educated.

5:19 ¶ 266 in Clinic D - FGD

PARTICIPANT 46: Because every day on the TV and whatever, there is Hansa, there is but there is no PrEP.

5:20 ¶ 262 in Clinic D - FGD

PARTICIPANT 51: What he said is here? in Orange Farm they listen to SAFM? a lot, like she was saying they use media, but not, they use it once, if they can get a slot maybe once every week, maybe let us say every Tuesday they know at a certain time there will be talking about PrEP, then they are going to listen.

5:22 ¶ 247 in Clinic D - FGD

PARTICIPANT 50: Okay most of our parents listen to the radio. Youth, you will find them Facebook, You Tube, you will find them there, yes

6:14 ¶ 29 in Clinic E - FGD

PARTICIPANT 55: I think we also need to inform our communities on information about PrEP, we teach them, put posters everywhere, although, the only thing, especially the

department, the only thing they will be complaining the whole day, so if we have posters everywhere, maybe PrEP, having the information, then maybe.

6:19 ¶ 48 in Clinic E - FGD

PARTICIPANT 53: Like the client person, even the family, as she was saying, if we can have the advertise or even like the Mina programme, it is all over, even TV, you can even see, so the like nowadays they are coming because they see everything. Like if we can have that project, they are talking, they are advertising on radio, TV, pamphlets, so even the parents they can have their kids to understand, not to be our responsibility to teach always. Yes.

6:37 ¶ 167 in Clinic E - FGD

PARTICIPANT 55: In we do have a poster, only a poster, we do not have pamphlets

6:38 ¶ 156 in Clinic E - FGD

PARTICIPANT 54: It makes it easier when they come in they already know, even if you have forgotten maybe to talk about it, then they will be like oh, also I heard they are talking about PrEP or maybe have the pamphlets because we also have pamphlets here, so they will be like they talked about this, can tell me more about it and all that.

○ Strategies to improve PrEP uptake and retention: 4.2 Strategies for information dissemination. Information regarding side-effects

5 Quotations:

3:28 ¶ 235 in Clinic B - FGD

PARTICIPANT 38 To erase or to minimise the impact of negative attitude both ways. Its only the information, giving of the information, only very, the drug, all the side effects, the management and how long are they going to last, that will only tool that will be able to reduce the attitudes.

3:68 ¶ 182 in Clinic B - FGD

PARTICIPANT 31: With the side effects usually its initiation, we try to give them health education which they must expect this minor side effect when they are taking PrEP, they only usually last for a week or two. So they should really expect them to last for a week or two, after that they are going to be fine. So maybe if we tried to emphasise that education and initiation maybe. They won't stop taking the medication.

5:6 ¶ 45 in Clinic D - FGD

PARTICIPANT 46: No, I think you know if we have given them a thorough health education regarding side-effects, I do not think it will be a problem because now from the history that I got from my clients that the side-effects are not that severe, it is not something that we will worry about because it is not only this treatment that has the side-effect, so I think that if we make sure that each and every client who is going to be initiated on PrEP and then knows about the side-effects, nothing will go wrong.

5:7 ¶ 47 in Clinic D - FGD

PARTICIPANT 49: I actually feel that I am with her definitely because if we actually provide them with the proper knowledge, they will be knowing what to expect. You cannot be dealing with something that you do not even know how to deal with it. Yes, but knowing very well that this is what is actually expected from this particular treatment that I am taking, you will actually be rest assure

5:52 ¶ 49 in Clinic D - FGD

PARTICIPANT 49: So if we could provide them with thorough education on initiation, then we will be actually like really helping them to understand the path they are actually choosing to take. So as far as side-effects are concerned they will be knowing what to expect and how to deal because basically you will be telling them, this is what you should expect and this is how you can actually manage it at home. So they will be quite well informed about this particular product?.

○ Strategies to improve PrEP uptake and retention: 4.3 Strategies for information dissemination. Information dissemination to enhance access to PrEP

3 Quotations:

5:49 ¶ 184 in Clinic D - FGD

PARTICIPANT 51: For example mostly that I have observed, in C4 when we initiate that is where we get [like, it is like when? we collect an index so that is where we get to know that, has your partner tested, bring your partner, so then also in that manner, you also recruit, also you give health education to the partner and then that partner will bring his or her partner and that is where you give education to say that here we have this tablet and then maybe if he or she agrees to go and test we…

6:33 ¶ 110 in Clinic E - FGD

PARTICIPANT 53: If the person nothing can help teaching, giving them health education, but you need to test and pull blood because the treatment that you are going to take, we need to check also your kidneys, so we must give them the health education, although with

some they are having phobia of injections, as she is saying, you will teach and teach and teach and then they will understand, when it comes for the injection, she leaves everything.

6:34 ¶ 177 in Clinic E - FGD

PARTICIPANT 53: To me I think it is more health education and to teach them there is a resistance, maybe they will be scared to like, if they feel like stopping the medication they will just stop and then if she wants to come back, she comes back, so if there is just more in health education.

○ Strategies to improve PrEP uptake and retention: 4.4 Strategies for PrEP. Convenient mode of administration

16 Quotations:

1:29 ¶ 112 in Clinic A - FGD

PARTCIPANT 29: If there is a way or if there is a certain PrEP method that can be used that has less side effects, that has less job of taking a pill daily. If for instance they could say there is a gum that you could eat and it would prevent you from HIV then let it be explored, but for as long as we are using this same method of and I am HIV negative, it is not going to work.

1:74 ¶ 296 in Clinic A - FGD

PARTICIPANT 28: Or maybe if it was injectable so that you only come once after every 3 months.

1:78 ¶ 300 in Clinic A - FGD

PARTICIPANT 28: You know if I would take it once then I am covered for 3 months, I would come. I would come back you know.

3:18 ¶ 156 in Clinic B - FGD

PARTICIPANT 31: The fact that PrEP is a pill that should be take every day, it’s a problem for our young girls, maybe if we could have PrEP in a form of injection or something else. Maybe the intake could improve.

3:19 ¶ 166 in Clinic B - FGD

PARTICIPANT 32: With the uptake because I don't believe the injection would be a monthly injection. Maybe two monthly or every three months. That could help

4:46 ¶ 160 in Clinic C - FGD

PARTICIPANT 40: If there is a way of having either one pill in three months or an injection, one injection in three months.

4:51 ¶ 400 in Clinic C - FGD

PARTICIPANT 44 If possible that they can manufacture an injection or pill, if that is possible.

4:52 ¶ 394 in Clinic C - FGD

PARTICIPANT 40: Like I said, if you can have one injection in three months.

5:10 ¶ 59 in Clinic D - FGD

PARTICIPANT 51: Okay, on that note because starting from my experience with them while I recruit them, they have a problem, the PrEP is the pill and they keep saying to me if there was an injection it was something [that they come really? , it is a once-off , like the Depo that they use for the prevention that would have worked. So what we can do for the fact, the ARV part of it [we can once-off injection so they are more comfortable about it?

5:24 ¶ 85 in Clinic D - FGD

PARTICIPANT 46: Yes it is true, that we can maybe get it. I think it can be 50, 50 because there are some who prefer the injection and then there are some who prefer oral because now when you go that side of family planning, it is where you get their views regarding this oral you know. They are saying Sister when you interview that client to say why are you here today, they will tell you that, Sister I am here for my family planning and then I am on injection. So for those maybe we can reach those who are using the injectable rather than giving them to go and swallow this pill each and every day.

5:25 ¶ 93 in Clinic D - FGD

PARTICIPANT 49: I believe if we provide them with a choice or options it will actually work for them. Now whereby actually they have a tablet, we offer them, and then we offer them injection as well. They are actually at liberty of making a right choice for them that is going to be actually you know, something that they could actually go on for a long time with. Unlike it is actually like I mean no option, it is only tablet, whereby if I am actually like I mean I am having this particular choice, I would actually prefer injection. There is none. It is [only like the tablet. So providing with a second option of injection, it will actually increase our chances of covering everybody that we look over as providing

5:26 ¶ 97 in Clinic D - FGD

PARTICIPANT 50: I think if it is possible [there maybe? could be a pill, like the Pill, that they take only once a month [for protection for some reason, a pill that they can take only

per month or two months if possible, so I think that will [help because some of them then? complain Participant 1 said, I do not want to take pills every day.

5:41 ¶ 227 – 229 in Clinic D - FGD

PARTICIPANT 49: Also the other option can actually be implant. How about that?

INTERVIEWER: So it can make a PrEP an implant like. Like implant.

PARTICIPANT 49: And then you know you are actually safe for the whole 3 years.

5:42 ¶ 225 in Clinic D - FGD

PARTICIPANT 50: I would go back to pills like if there could be one or two pills and the injection like it could be like 50, 50. If you do not want the pills, you can take the injection, if you do not want the injection you can take the pills, so in that way I think that

6:6 ¶ 17 in Clinic E - FGD

PARTICIPANT 55: Yes, I think she is correct. Taking a pill every day, if it was injected or maybe let us say for like just get it once in three years, I think maybe it is going to be better in that way.

6:35 ¶ 173 in Clinic E - FGD

PARTICIPANT 54: I always like to think the only way is injection, injectables, injectables, because I feel like most of the patients, you are asking them why they are not taking, oh I do not want pills, so okay what can help. Is there an injection maybe and all of that, so I also feel like an injection will also help.

○ Strategies to improve PrEP uptake and retention: 4.5 Strategies for youth. Peer encouragement to take PrEP

1 Quotations:

6:30 ¶ 102 in Clinic E - FGD

PARTICIPANT 54: I wanted to say, like she said also about peer pressure, it is also that because if you have one patient sitting outside and one is being taught about PrEP and then they go out and they did not get PrEP, they are also wanting to know why did I not also get PrEP, so they want to get it. So it is also about what the next person or the friend has gotten, you understand, so if they are together maybe supporting each other they will be able to take PrEP because of now they are having another person doing the same thing, all their friends taking it, so now if the friend is like no, you are taking ARV’s and all of that, obviously they are going to leave that and say no I am done with this.

○ Strategies to improve PrEP uptake and retention: 4.6 Social strategies. Strategies for destigmatisation and non-discrimination

2 Quotations:

5:23 ¶ 245 in Clinic D - FGD

PARTICIPANT 50: I want re-ad what Participant 1 said before about social media. I think if everyone knows about PrEP, I will not be shy to take PrEP because my parent knows about PrEP, even my friends? know about PrEP, so in that way I think the stigma of HIV family and friends it will not be there.

6:18 ¶ 44 – 46 in Clinic E - FGD

PARTICIPANT 56: To me a stigma nowadays it is no more like before, yes, if they can get like education, they will understand that okay it is an ARV but you are taking the preventative, it is not the ... what you call ...

INTERVIEWER: Is the ARV?

PARTICIPANT 56: Yes it is not the ARV per se yes you can preach that...

○ Strategies to improve PrEP uptake and retention: 4.6 Social strategies. Strategies for parents

4 Quotations:

5:12 ¶ 61 in Clinic D - FGD

PARTICIPANT 48: But on the other side, parents; it is not like they are not being supportive as such, because they nothing about it. If they were informed maybe they can be very much supportive.

5:15 ¶ 63 in Clinic D - FGD

PARTICIPANT 50: I think we should educate the parents because mostly what I saw is that parents come here, so I think when they are [in your clinic that is when I think there may be a talk and tell them about PrEP so that they cannot be surprised when their kids are taking PrEP.

6:9 ¶ 23 in Clinic E - FGD

PARTICIPANT 54: The only thing is just to give education, teach, teach, teach, because I do not really think that there is any other thing that could be done, they also need information on how these things work, because now we are only giving the information only to the girls, but we are not giving information to the mother

6:20 ¶ 52 in Clinic E - FGD

PARTICIPANT 54: Okay, with that I would say it is also just education, education at this point, because then if they have the education I do not really think they would stop their kids from taking the PrEP and everything, so just -
