# Supplementary material for: Healthcare systems barriers and strategies for pre-exposure prophylaxis utilization amongst young females in Gauteng province: Registered nurse’s perspectives
Source: PLoS One. 2025 Jun 12;20(6):e0294182. doi: 10.1371/journal.pone.0294182 (PMC12161546; doi:10.1371/journal.pone.0294182)
Supplement: S2 File — Focus group discussion guide for professional nurses. (DOCX) [file pone.0294182.s002.docx]

| 1. **FOCUS GROUP DISCUSSION GUIDE FOR PROFESSIONAL NURSES.** |
| --- |

**Word of welcome and appreciation to participate in the study**

**Sub-section A1. Descriptive data of the participants**

**Age:**

| 30 and below |  | 31-50 |  | 51-60 |  | Above 60 |  |
| --- | --- | --- | --- | --- | --- | --- | --- |

**Years of experience in PrEP:**

| 12 months or less |  | More than12 Months |  | None |  |
| --- | --- | --- | --- | --- | --- |

**Level of education:**

| Diploma |  | Degree |  | Honours |  | Masters |  | PhD |  |
| --- | --- | --- | --- | --- | --- | --- | --- | --- | --- |

**Occupation category:**

| Professional Nurse |  | Senior Professional nurse |  | Clinic manager |  |
| --- | --- | --- | --- | --- | --- |

**Marrital status:**

| Married |  | Single |  | Divorced |  | Separated |  | Widowed |  | Engaged |  | In a relationship |  |
| --- | --- | --- | --- | --- | --- | --- | --- | --- | --- | --- | --- | --- | --- |

| Other (Please specify) | ………………… |
| --- | --- |

**Gender:**

| Female |  | Male |  |
| --- | --- | --- | --- |

| Other (Please specify) | ………………… |
| --- | --- |

**Sub-section A2. Recap on:**

- **Study topic**
- **Study purpose**

**Sub-section A3. Questions to Be Asked During Discusion:**

- Have you been trained in PrEP?
- What do you know about Pre-Exposure Prophylaxis (PrEP)?
- What are the advantages amd disadvantages of taking PrEP
- Who is eligible or not for PrEP?
- Who do you offer PrEP health education to?
- What health education are you documenting for PrEP?
- What is your expirience in prescribing PrEP and what encourages or discourages you to?
- What is your expirience with young females in relation to PrEP?
- What may encourages or discourage patients to take PrEP?
- What may discourages patients to be complient with PrEP prescription?
- What are patient or health system or health professionals factors influencing PrEP uptake and retention?
- What may causes patients to stop PrEP
- How can we influence patients or support them to take and continue with PrEP ?
- Advise the Department of Health on how best PrEP can be implimented to increase the uptake and retention?
